# Resistance training reduced luteinising hormone levels in postmenopausal women in a substudy of a randomised controlled clinical trial: A clue to how resistance training reduced vasomotor symptoms

**DOI:** 10.1371/journal.pone.0267613

**Published:** 2022-05-26

**Authors:** Sigrid Nilsson, Moa Henriksson, Emilia Berin, David Engblom, Anna-Clara Spetz Holm, Mats Hammar

**Affiliations:** 1 Obstetrics and Gynaecology, Division of Children’s and Women’s Health, Department of Biomedical and Clinical Sciences, Linköping University, Linköping, Sweden; 2 Division of Cell Biology, Department of Biomedical and Clinical Sciences, Linköping University, Linköping, Sweden; Prince Sattam Bin Abdulaziz University, College of Applied Medical Sciences, SAUDI ARABIA

## Abstract

**Background:**

Vasomotor symptoms (VMS) are common around menopause. Menopausal hormone therapy is the most effective treatment for VMS. Physical exercise has been proposed as an alternative treatment since physically active women have previously been found to experience fewer VMS than inactive women. In our randomised controlled trial on resistance training to treat VMS, sympoms were reduced by 50% in the intervention group compared with the control group.

**Objectives:**

To propose a mechanism to explain how resistance training reduced VMS and to assess if luteinizing hormone (LH) and follicle stimulating hormone (FSH) were affected in accordance with the proposed mechanism.

**Trial design and methods:**

A substudy of a randomized controlled trial on 65 postmenopausal women with VMS and low physical activity who were randomised to 15 weeks of resistance training three times per week (n = 33) or to a control group (n = 32). To be regarded compliant to the intervention we predecided a mean of two training sessions per week. The daily number of VMS were registered before and during the 15 weeks. Blood samples were drawn for analysis of LH and FSH at baseline and after 15 weeks.

**Results:**

LH decreased significantly in the compliant intervention group compared with the control group (-4.0±10.6 versus 2.9±9.0, p = 0.028 with Mann-Whitney *U* test). FSH also decreased in the compliant intervention group compared with the control group, however not enough to reach statistical significance (-3.5±16.3 versus 3.2±18.2, p = 0.063 with Mann-Whitney *U* test). As previously published the number of hot flushes decreased significantly more in the intervention group than in the control group but there was no association between change in LH or FSH and in number of VMS.

**Conclusions:**

We propose that endogenous opiods such as β-endorphin or dynorphin produced during resistance training decreased VMS by stimulating KNDγ-neurons to release neurokinin B to the hypothalamic thermoregulatory centre. Through effects on KNDγ-neurons, β-endorphin could also inhibit GnRH and thereby decrease the production of LH and FSH. The significanty decreased LH in the compliant intervention group compared with the control group was in accordance with the proposed mechanism.

## Background

Vasomotor symptoms (VMS) including hot flushes and night sweats are reported by most women around menopause [1, 2]. VMS have a median duration of 5–7 years but may persist for more than 15 years [3] and may interfere with daytime activities, sleep, and quality of life [1, 2]. Menopausal hormone therapy (MHT) is the most effective treatment for VMS. In women initiating MHT below 60 years of age or within ten years from menopause, all-cause mortality is reduced [4] but in women initiating MHT later there is a higher absolute risk for coronary heart disease, stroke, venous thromboembolism, and dementia [5]. In women with a history of breast cancer MHT is contraindicated [6]. Therefore, alternatives to MHT for VMS are needed.

Observational studies have reported fewer VMS in physically active than sedentary postmenopausal women [7–9]. Intervention studies on the effect of physical activity on VMS show conflicting results, possibly because of low compliance, low intensity, and high drop-out rate [10, 11]. In our randomised controlled trial on the effect of 15 weeks of resistance training on VMS, symptoms were reduced by 50% in the intervention group compared with the control group [12]. The present substudy aimed to propose a mechanism to explain why VMS decreased during resistance training. We also aimed to assess if luteinizing hormone (LH) and follicle stimulating hormone (FSH) were affected during the 15 weeks in accordance with the proposed mechanism. A secondary aim was to assess if there was an association between the decrease in LH and FSH and the decrease in VMS.

VMS in postmenopausal women are probably caused by thermoregulatory instability. The preoptic area (POA) of the anterior hypothalamus is the primary autonomic thermoregulatory area with warm and cold-sensitive afferent neurons. The POA is capable to initiate effector responses to maintain normal core temperature in spite of changes in the surrounding temperature or the endogenous heat production [13, 14]. Within the thermoneutral zone, minor changes in core temperature are regulated by constriction or dilation of peripheral arteries, mediated by autonomic noradrenergic innervation [15–17]. Changes in core temperature overriding the limits of the thermoneutral zone activate more powerful heat dissipation responses such as cutaneous vasodilation and sweating, mediated by cholinergic neurotransmission [18]. This is experienced as a sudden feeling of heat and sweating during VMS [16, 19]. Calcitonin gene-related peptide (CGRP) may mediate cholinergic vasodilation and activation of sweat glands causing the VMS [20, 21]. Blockge of CGRP in a mouse model eliminated VMS [22]. Also, serum CGRP increases during VMS in women [23].

Estrogen acts stabilizing on the thermoregulatory centre and the decrease in estrogen around menopaus leads to a narrowing of the thermoneutral zone [16, 19]. In women with VMS, smaller increases in core temperature activate the heat dissipation responses compared with asymptomatic women [24].

Cell bodies in the POA of the hypothalamus pulsatily secrete gonadotropin releasing hormone (GnRH) into the portal circulation leading the pituitary to produce and release the gonadotropins Luteinizing Hormone (LH) and Follicle Stimulating Hormone (FSH). LH and FSH regulate the ovaries to follicular growth, production of estrogens, and ovulation. Estrogens exert negative feedback on GnRH-release from the POA. After menopause, the amplitude and frequency of LH and FSH secretion increase due to reduced negative feedback from estrogens and inhibin.

Some studies have shown that VMS synchronize with LH pulses [25, 26] but LH pulses do not cause VMS since women with suppressed gonadotropins, either through hypophysectomy or treatment with GnRH analogues, are still affected by VMS. Nor do GnRH pulses cause VMS, since women with Kallman’s syndrome experience VMS after estrogen withdrawal despite their absence of functional hypothalamic GnRH neurons [27, 28].

Kisspeptin/Neurokinin B/Dynorphin-neurons (KNDγ-neurons), originating from the infundibular nucleus of the hypothalamus [29], mediate the negative feedback between estrogens and GnRH via the neuropeptide kisspeptin [30]. Estrogen affects KNDγ-neurons both directly by receptor-binding and indirectly by increasing the production of β-endorphin [31] which in turn probably acts on KNDγ-neurons. Neurokinin B and dynorphin act autoregulatory, affecting kisspeptin secretion. Dynorphin is an endogenous opioid peptide that inhibits kisspeptin secretion via the κ opioid receptor [32]. Neurokinin B stimulates kisspeptin production via the neurokinin-3 receptor (NK3R) [33, 34]. KNDγ-neurons are also involved in the central thermoregulation [35, 36], projecting to heat dissipation nuclei in the POA [37] where neurokinin B is released [38]. Thus, KNDγ-neurons constitute a possible link between the fall in estrogen during the menopausal transition and changed thermoregulation.

KNDγ-neurons are hypertrophied in postmenopausal women [39, 40]. Furthermore, the gene expression of dynorphin mRNA is downregulated while the expression of both kisspeptin and Neurokinin B are upregulated in postmenopausal women [32, 41]. These phenomena are likely consequences of estrogen withdrawal, since they also have been observed in monkeys after ovariectomy and can be reversed with exogenous estrogens [29]. Activation of a type of KNDγ-neuron (Kiss1ARH) in mice triggered heat-dissipation with vasodilation and reduced core-body temperature (resembling VMS), an effect that was enhanced after ovariectomy [38].

Neurokinin B release in the thermoregulatory centre in the POA could cause thermoregulatory instability during VMS in postmenopausal women [38]. Injection of a selective NK3R agonist (senktide) into POA of rats enhanced skin vasomotion and reduced the inner core temperature [30]. In humans, intravenous infusion of a NK3R agonist in postmenopausal women induced the characteristics of VMS, with enhanced cutaneous blood flow and interestingly also a peak of LH in plasma [42]. Furthermore, NK3R antagonist treatment has been found to reduce VMS in women [43].

After menopaus a decreased negative feedback by estrogen directly on KNDγ-neurons and reduced production of endogenous opioids such as dynorphin and β-endorphin [32, 44] in the central nervous system (CNS) can explain the upregulation of NKB. β-endorphin in CNS is affected by estrogen through activation of ERα on hypothalamic neurons, increasing the production of Pro-opiomelanocortin which can be cleaved to β-endorphin [45, 46]. Consequently, the concentration of β-endorphin in cerebrospinalfluid decreases after menopause [47]. A pilot study using κ-agonists to enhance the dynorphin activity within the KNDγ-neuron network has also showed promising results in treating VMS [48].

β-endorphin is both released in CNS and the peripheral circulation during activation of large muscle groups, e.g. during resistance training [49, 50]. Since β-endorphin has been suggested to stabilize thermoregulation we proposed that resistance training may decrease VMS by an induction of β-endorphin production in CNS. Based on the previously discussed information, the proposed mechanisms behind VMS and the role of resistance training are summarized in Fig 2A–2D.

Functional hypothalamic amenorrhea (FHA) is a state of hypogonadotropic hypogonadism caused by weightloss, stress, or excessive exercise [51, 52]. These stressors may induce β-endorphin production in CNS which suppresses GnRH pulsatility through effects on KNDγ-neurons. Administration of the opioid antagonist naltrexone in women with FHA increased the LH pulsatility in plasma and restored the menstrual cycle [53], suggesting that increased opioid signalling at least contributes to the hypogonadism in FHA. Despite their low levels of estrogens, women with FHA do not experience VMS [54]. The absence of VMS in FHA is probably explained by the enhanced opioid activity in the hypothalamus, which except for impairing GnRH pulsatility also could stabilize the thermoregulation. If the opioid signaling could be restored in postmenopausal women through resistance training similar to what is seen in athletes with FHA, VMS could possibly be avoided.

In male rats intensive physical activity for six months reduced the expression of GnRH genes via increased dynorphin gene expression in KNDγ-neurons and subsequently, LH levels in plasma were decreased [55]. Thus, decreased levels of LH could be an effect of enhanced opioid signalling on KNDγ-neurons. Accordingly, LH in plasma was reduced in both amenorrhoic [56] and eumenorrheic female endurance athletes [57], suggesting that a similar mechanism was involved.

**Fig 1:**
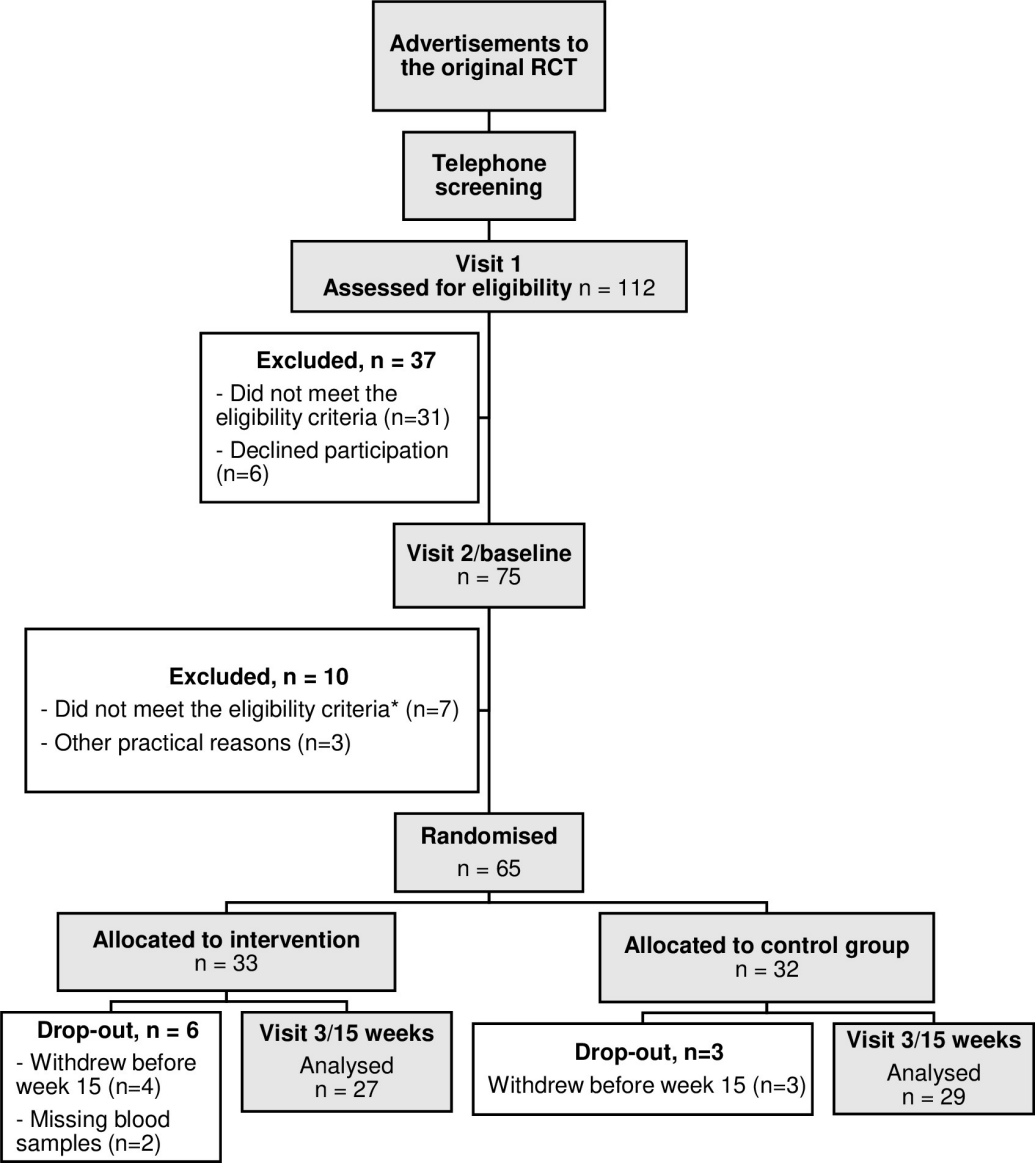
A flowchart showing the number of women participating in the RCT. Flowchart according to Consort showing the number of women included, randomized, and followed up in a 15 week Randomized Controlled Trial (RCT) of resistance training as a treatment for vasomotor symptoms and analyzed for LH and FSH levels. * excluded due to too few hot flushes according to diary during the two weeks between the screening visit (visit 1) and the baseline visit (visit 2).

As previously mentioned, we have performed a RCT showing that VMS decreased by 50% in postmenopausal women randomised to resistance training compared with the control group [12]. The hypothesis for this current substudy of the RCT, leading to its objective, was that LH and FSH would decrease in postmenopausal women with VMS during 15 weeks resistance training, possibly due to enhanced opioid activity affecting the KNDγ-neurons. If so this could suggest an explanation why VMS were reduced in these women. These proposed mechanisms are illustrated in Fig 1D.

## Material and methods

### Trial design and participants

This was a substudy of an open, parallel-group Randomised Controlled Trial (RCT) conducted at the Department of Obstetrics and Gynaecology at the University Hospital of Linköping, Sweden. The trial was preregistered as a clinical trial in the database ClinicalTrials.com with ID: NCT01987778 and with a published study protocol [58].

The study population (n = 65) consisted of postmenopausal women with VMS and low physical activity recruited between 2013 and 2017. Women in the original study were followed up until 2019. Through advertisements in the local newspapers and at the University Hospital in Linköping, women were informed to call or send e-mail to the clinic if they were interested in participating. An initial telephone screening about physical activity habits and menopausal symptoms was performed, followed by a screening visit to the outpatient clinic. After oral and written informed consent the visit to the the department of obstetrics and gynecology, University Hospital of Linköping included physical examination, blood sampling, and control of inclusion and exclusion criteria which are summarized in Table 1, and described in more detail elsewhere [12].

**Table 1:** Inclusion and exclusion criteria for the women included in the trial.

Inclusion criteria	Exclusion criteria
• Woman at least **45 years old** • Postmenopausal status ≥ 12 months without menstruation FSH ≥ 20 IU/L for women with ◾ intrauterine device with progestogens ◾ hysterectomy • ≥ 4/day or 28/week moderate-severe VMS • Good health status ○ Physical ability to perform a resistance training program	• Menopausal hormone therapy within the last three months • Physically active per definition ○ >225min/week of mixed physical intensity ○ >75min/week of moderate to vigorous physical intensity • Medical condition with ○ potential to affect the number of hot flushes ○ contraindication for resistance training • Unstable dose of SSRI, SNRI or other medications with potential effects on VMS • Haemoglobin <110g/L • Systolic blood pressure >160mmHg • Diastolic blood pressure >100mmHg

FSH = Follicle-stimulating hormone; VMS = vasomotor symptoms; SSRI = Selective serotonin reuptake inhibitors; SNRI = Serotonin and noradrenaline reuptake inhibitors.

After the screening visit women registered VMS and physical activity in a diary for two weeks. The diary registration was controlled at a visit two weeks later when eligible women with a mean of at least four moderate to severe hot flushes per day were randomized.

### Randomization

We used block randomisation in a 1:1 allocation. An independent statistician created the allocation sequence using a computer-based random number generator (Stata 13.1, StataCorp LP, Texas, USA). A label stating if the woman was to belong to the intervention or control group was placed in opaque, sealed envelopes numbered from 1 to 65. The envelope was opened by the woman in front of the physician at the randomization visit and the label was saved in the Case Record Form. All individuals involved in data analysis were blinded for group allocation until the statistical analyses of the results were completed.

### Intervention

The women were randomised to either a supervised resistance training program for 15 weeks or a control group and were asked to fill in a VMS diary throughout the 15 weeks. The intervention was a three day/week whole body resistance training program supervised by a physiotherapist and described in more detail elsewhere [58] In short, the 15-week resistance training program consisted of eight exercises performed in two sets with two minutes rest between sets. The exercises were chest press, leg press, seated row, leg curl, latissimus dorsi pulldown, leg extension, crunches, and back raises. Six exercises were performed in seated resistance machines and two were body-weight exercises. The seated exercises were performed with 15–20 repetitions (week 1–3) to minimize risk of injury and 8–12 repetitions from week 4–15. Exercise sessions were preceded by 7–10 minutes warm-up and finished with dynamic and static stretching. Loads were individually set by the physiotherapist after a test of muscle strength (eight-repetition maximum) and thus we used individually tailored programs regarding weights but predecided number of repetitions based on strength measurements at baseline and after three weeks. To be considered compliant according to the protocol we defined participation on average at least twice a week. The participants in the control group were instructed to keep their low level of physical activity for 15 weeks which could be verified by means of diaries and a validated questionnaire at the 15 weeks follow-up. After 15 weeks all women came to a third visit for follow-up, blood sampling and answering questionnaires. (See Flowchart Fig 1).

### Outcomes

In this substudy of the previously published RCT [12] the primary outcome was change in LH and FSH levels between baseline and after 15 weeks. The secondary outcome was the correlation between changes in LH and FSH and changes in VMS.

### Biochemical assays

Fasting venous blood samples were drawn and collected in EDTA vacutainers (BD AB, Sweden) at baseline and at the week-15 visit. Plasma was aliquoted after centrifugation at 1500 G and stored for future analyses at -70°C. All plasma samples were then thawn and analysed in one batch. LH and FSH were analysed as previously described [59] according to routines in an accredited laboratory of clinical chemistry (ISO/IEC 17025; Linköping University Hospital, Linköping, Sweden).

### Ethical considerations

The RCT was conducted in accordance with Good Clinical Practice–except from monitoring only during the study—and the Declaration of Helsinki, with oral and written informed consent before participation. The Regional Ethical Review Board in Linköping approved the research protocol, including analyses of LH and FSH, for the RCT (2013/285-31).

### Data analyses and statistical methods including sample size calculation

**Fig 2:**
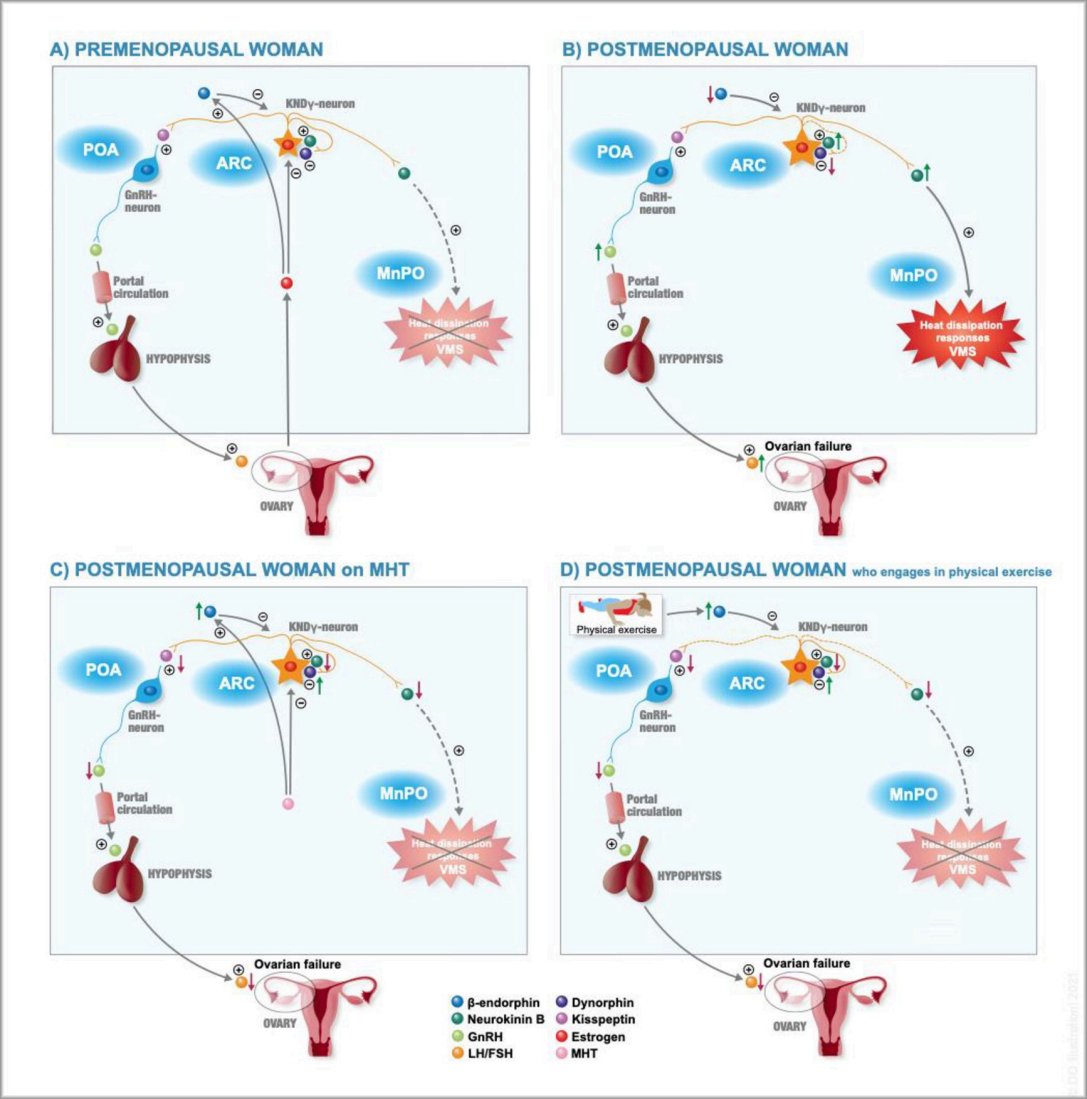
**A-D**: Illustrations to the possible cause of vasomotor symptoms and how Menopausal Hormone Therapy (MHT) and physical exercise may relieve them. A PREMENOPAUSAL WOMAN: The GnRH cell bodies in the preoptic area (POA) of hypothalamus constitute the central regulation of reproduction. The pulsatile GnRH secretion into the hypophyseal circulation triggers the production and release of the gonadotropins Luteinizing Hormone (LH) and Follicle Stimulating Hormone (FSH). Since GnRH neurons do not express steroid receptors, evidence suggests that indirect steroid feedback is mediated by Kisspeptin/Neurokinin B/Dynorphin (KNDγ) neurons. Via the neuropeptide kisspeptin, the KNDγ neurons stimulate both the GnRH pulse amplitude and frequency. Except their steroid-responsiveness and involvement in reproduction, KNDγ neurons also project to heat dissipation neurons in POA and its Median Preoptic Nucleus (MnPO), mainly via the neuropeptide Neurokinin B (NKB). The MnPO neurons constitute the primary autonomic thermoregulatory area, capable to initiate effector responses in order to stabilize the inner core temperature in a changing ambient environment. Since estrogen acts stabilizing on the thermoregulatory center by widening the thermoneutral zone, premenopausal women are less afflicted by vasomotor symptoms (VMS). B POSTMENOPAUSAL WOMAN: In postmenopausal women, the ovarian failure leads to reduced negative feedback from sex steroids, which is associated with increased KNDγ neuron activity. The gene expression of dynorphin mRNA is downregulated while the expression of both kisspeptin and NKB are simultaneously upregulated. The reduced levels of centrally produced β-endorphin further increase the KNDγ neuron activity causing hypertrophy of the nucleus. Consequently, there is an instability in the thermoregulatory center in MnPO, narrowing the thermoneutral zone, which in turn facilitates inappropriate activation of thermoregulatory effectors for heat dissipation, clinically experienced as VMS. Moreover, the increased kisspeptin expression stimulates GnRH neurons which both enhances the gonadotropin pulse frequency and amplitude from the pituitary. C POSTMENOPAUSAL WOMAN ON MHT: Endogenous estrogen and menopausal hormone therapy (MHT) modulate the thermoregulatory and reproductive functions indirectly via the hypothalamic opioid system. Both β-endorphin and dynorphin signaling lower the KNDγ neuron activity, leading to reduced levels of the stimulatory neuropeptides kisspeptin and NKB. This inhibits the pulsatile GnRH release to the portal circulation, and stabilizes the thermoregulatory center in MnPO by a widening of the thermoneutral zone, therefore decreasing VMS. D POSTMENOPAUSAL WOMAN WHO ENGAGES IN PHYSICAL ACTIVITY: Peripheral estrogen concentrations positively correlate with the central opioid tone. Thus, postmenopausal women may have a relative deficiency of hypothalamic endogenous opioids due to their ovarian failure. Moderate to intense physical activity may increase the production of hypothalamic opioids and thereby lower the KNDγ neuron activity in a similar mechanistic way as for endogenous estrogen or MHT. Therefore, physical activity could be an alternative to MHT for postmenopausal women.

Data distribution determined choice of statistical method and if the presented data were expressed as mean ± standard deviation (SD) or median (25th and 75th percentile). During statistical analyses, both the intention to treat (ITT) intervention group according to the flow chart Fig 2 and the compliant intervention group that participated in resistance training on average at least twice weekly (per protocol, PP) were accounted for. Independent statistical analyses between the groups at the different time points (baseline and 15 weeks) were performed using the parametric T-test or non-parametric Mann-Whitney *U* test. Dependent analyses within the groups across time were performed using the parametric T-test for paired samples. Absolute changes from baseline to the week-15 visit are presented and compared between groups using the non-parametric Mann-Whitney *U* test. There were few dropouts and hence few missing data (two out of 58). The participants with no follow-up data were not statistically different at baseline from the analysed participnats regarding age, Body Mass Iindex, time since menopause, LH or FSH values. Missing data were not imputed in the analyses.

We considered results with p<0.05 statistically significant. The data analyses were performed using SPSS v.26.0 (IBM, Portsmouth, UK).

The sample size calculation for the original RCT was based on results from a pilot study including the first 16 participants in the trial. Forty participants, 20 in each group, were needed to detect a 50% difference in moderate and severe hot flushes with 80% power and an expected dropout rate of 20%. A 50% decrease in hot flushes has been considered a clinically significant change for women. We expanded the inclusion to 60 participants to increase the power of secondary outcome variables. Thus for the present substudy we performed no further inclusion of participants.

## Results

The participant flow is shown in Fig 1 including numbers of women randomized to the intervention and control group, received the intended intervention, and were analyses regarding serum levels of LH and FSH at baseline and after 15 weeks. There were no harms noted in any group over the 15 weeks and no women withdraw due to for example musculoskeltal overuse symptoms.

Individual characteristics at baseline and group comparisons are presented in Table 2. The women of the control group and intervention group were middle-aged (55±5 and 56±5 years, respectively) with a median postmenopausal time of 33 (14–70) months in the control group and 36 (16–84) months in the intervention group. The participants were normotensive, and on average overweight according to BMI (27.9±4.0 and 26.7±3.6 kg/m^2^, respectively). There were no significant differences in baseline characteristics, anthropometric values, or LH at baseline whereas FSH was significantly higher in the control group but both groups had typical postmenopausal levels.

**Table 2:** Baseline characteristics of the original randomisation groups.

*Variables*	Intervention group (n = 29)	Control group (n = 30)	P-value
Age (years) ^1^^,^^2^^,~^	56±5	55±5	0.919
Menopausal time (months)^§^^,^^1^^,^^3^^,^^¤¤¤^	36(16–84)^¤^	33(14–70)^¤¤¤^	0.583
Weight (kg) ^2^^,~^	76.1±12.0	72.3±11.5	0.218
BMI (kg/m^2^) ^2^^,~^	27.9±4.0	26.7±3.6	0.237
WC (cm) ^2^^,~^	92.1±12.5	88.8±12.9	0.322
SBP (mmHg)^2^^,~^	131±14	128±17	0.556
DBP (mmHg) ^2^^,~^	78±7	78±10	0.883
Testosterone (ng/mL)^3^^,^^§^	0.45(0.25–0.61)	0.54(0.34–0.96)	0.139
SHBG (nM) ^3^^,^^§^	33.0(22.7–41.6)	31.2(20.1–42.7)	0.883
LH (IU/L))^2^^,~^	37.1±13.4	39.3±15.8	0.576
FSH (IU/L) ^2^^,~^	63.3±20.0	84.2±38.4	0.013*

BMI = Body Mass Index; WC = Weight circumference; SBP = Systolic blood pressure; DBP = Diastolic blood pressure; SHBG = Sex-hormone binding globuline; LH = Luteinizing hormone; FSH = Follicle-stimulating hormone

^1^ Measured at baseline

^2^ Independent samples T-test

^3^ Mann-Whitney U-test

^¤^ n = 27

^¤¤^ n = 14

^¤¤¤^ n = 20; ~ Mean ± SD

^§^ Median (25^th^– 75^th^ percentiles)

**Fig 3:**
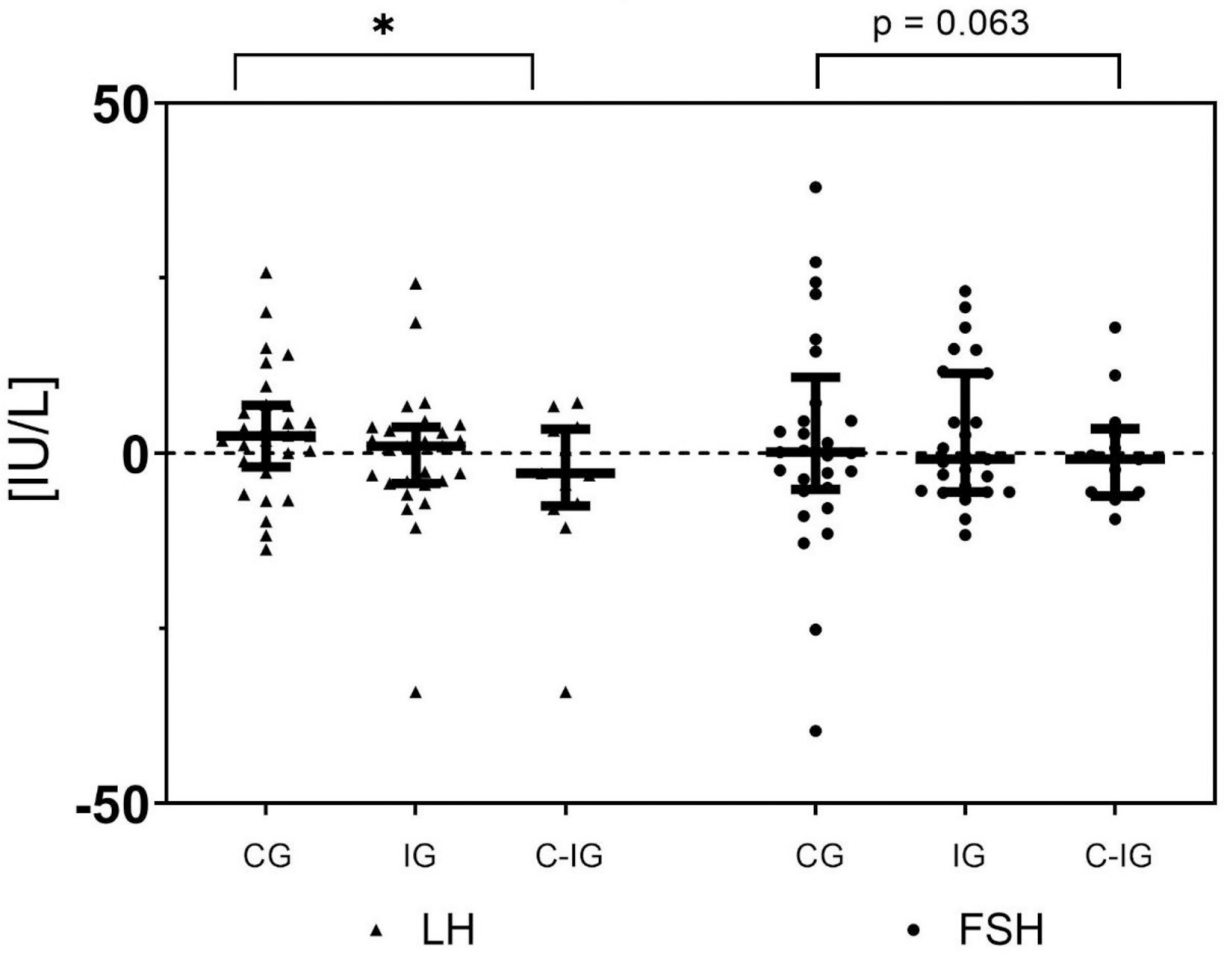
Scatter dot plot of the absolute levels of change in LH and FSH between the baseline and week-15 visit. Scatter dot plot of the absolute levels of change in LH and FSH between the baseline and week-15 visit. Separated plots are presented for each per-protocol defined group; control group (CG), intervention group (IG) and compliant intervention group (C-IG). The absolute change in LH over 15 weeks differed significantly between the compliant intervention group and the control group. A similar albeit non-significant trend was observed for FSH. * = p<0.05.

In the compliant intervention group LH decreased from 36.3 IU/L (+/- 12.0) to 34.4 IU/L (+/- 13.7) whereas LH increased in the control group from 39.3 (+/- 15.8) to 42.1 (+/- 15.4) IU/L. The difference in change between the groups was significant as shown in Fig 3 (Mann-Whitney *U* test, p = 0.028). The change in LH and FSH did not differ significantly between any other groups as illustrated in Fig 3 although the change in FSH from baseline to 15 weeks tended to be larger (p = 0.063) in the intervention group (-3.5 +/- 16.3 IU/L) than in the control group (3.2 +/- 16.2 IU/L).

Additionally, we analysed change from baseline to 15 weeks in LH and FSH in those women in the intervention group who had ≥ 50% decrease in hot flush frequency but neither LH nor FSH changed significantly and therefore did not correlate with the change in VMS over the 15 weeks.

## Discussion

In this substudy of a randomized controlled trial of 15 weeks resistance training in postmenopausal women with VMS, LH levels decreased significantly more in women compliant to the intervention compared with the control group, and FSH levels remained unchanged. This was partly in line with our hypothesis that resistance training would decrease mainly LH but also FSH in previously low-active postmenopausal women who increased their physical activity with a resulting reduction in VMS.

Previous research has shown that hypothalamic KNDγ-neurons are tightly linked to both VMS [30, 32, 37, 39, 40] and gonadotrophin production [60] and also could be affected by physical exercise [55–57]. A simultaneous decrease of LH, FSH and VMS following resistance training would be in accordance with the suggested mechanism. In Fig 2A–2D we summarize our suggested mechanisms regarding VMS after menopause and how MHT and physical exercise may affect VMS in postmenopausal women.

Whereas resistance training reduced LH levels as we hypothesized, we did not find the expected change in LH in participants who had a ≥ 50% reduction in hot flush frequency.

The discrepancy in our results may have multifactorial explanations. The gonadotropin pattern of secretion is divided into a basal background production interspersed with temporary pulses. Gonadotropin secretion can be modulated by several endogenous (e.g. endogenous opioids), exogenous including nutritional, and environmental cues, predominantly affecting either the background production or the pulse frequency or amplitude. Since each LH pulse corresponds to a GnRH release into the median eminence, measuring LH in plasma has for a long time been a validated method to evaluate the GnRH secretion [61]. Additionally, GnRH cannot be measured in peripheral blood samples [57].

Endurance athletes with hypothalamic amenorrhea are similar to postmenopausal women considering they both have low levels of estrogen and absence of normal menstruations. Low estrogen levels in endurance athletes are, however, accompanied by low levels of LH and most importantly absence of hot flushes, an observation that has been suggested to be caused by high levels of endogenous opioids [54, 56, 57]. By letting postmenopausal women engage in resistance training, a similar state with an enhanced central opioid tone as in endurance athletes may be induced, reducing the number of hot flushes as well as the LH levels. Intensity and volume of exercise, which were considerably lower in our trial than in endurance athletes with hypothalamic amenorrhea, may however be important for the potential to affect gonadotropin production and thermoregulation. In male rats, intense compared to moderate exercise during six months had distinct effects on GnRH gene expression and circulating levels of LH and testosterone [55]. The training intensity in the present study was sufficient to affect both LH levels and number of hot flushes in the per protocol compliant intervention group compared with the control group. The changes were, however, smaller than in endurance athletes with hypothalamic amenorrhea who did not experience hot flushes and had a greater reduction in gonadotropins [54, 56, 57].

The previously mentioned study using male rats also found that during exercise, NKB mRNA levels decreased and that dynorphin mRNA levels increased [55], opposing the changes observed during the menopausal transition [32]. These results support our hypothesis theoretically, although they remain to be confirmed in human subjects. Since NKB antagonism also has been suggested as a new treatment for menopausal hot flushes [43], and can be modulated by exercise [55], it is possible that resistance training may affect thermoregulation by antagonising the production of NKB and increasing the hypothalamic endogenous opioid activity via dynorphin, or both. The methodological design of this study, however, only allows us to speculate about this.

As mentioned, LH did not decrease in the participants who had a ≥50% reduction of hot flushes during the study, contrary to our hypothesis. The opposing effects on gonadotropin change across time for the “compliant” versus “VMS-reduction” groups could result from an individual heterogenicity with regard to the cause of hot flush reduction. Limited sample size restricted statistical analysis after stratification for both per protocol defined compliant to resistance training and ≥50% reduction in hot flush frequency. For the non-compliant participants in the intervention group, the hot flush reduction may be better explained by other mechanisms than KNDγ-neuron modulation. We can also speculate that belonging to the intervention group by itself could have a placebo-like effect on both hot flush frequency and severity.

Thus our study has a number of limitations. Blinding was impossible due to the intervention used but at least analyses of results were blinded regarding treatment until the calculations of the results and statistical analyses were completed. Since the gonadotropins are released in two secretion patterns, a basal background production interspersed with temporary pulses, a single measurement in plasma does not detect the gonadotropin pulsatility. Ideally, we would have performed multiple measurements of LH across time during one or several days. This methodology would have been more sensitive to impairment of GnRH pulsatility than merely measurements of LH concentration in plasma. An alternative would have been measurements of opioids in cerebrospinal fluid. Furthermore an association between LH changes in those women who had the most evident decrease in VMS, or an association between change in LH or FSH and VMS, would have been another support of our hypotheses than merely a decrease in LH in the compliant intervertion group. Ideally we should have measured dietary habits but we controlled for smoking habits (smokers usually have more VMS) and very few women were smokers. Furthermore there are drugs both prescribed and bought over-the-counter which might have affected the results but use of such drugs was an exclusion criterion not permitted in the study according to the protocol.

Research has led to important discoveries in the field of VMS, such as the involvement of the KNDγ-neuron network in the VMS pathoetiology [30]. These findings have subsequently resulted in several clinical trials with promising results [43, 48]. Nonetheless, there is still more to be known about the mechanisms behind VMS and why certain women are more afflicted than others by the symptoms. We suggest that resistance training is a safe alternative that will help many women through a distressing time with hot flushes and night sweats by desensitisation of the KNDγ-neuron network in hypothalamus via enhanced endogenous opioid production and reduced NKB stimulatory actions. The mechanisms behind these results are probably multifactorial, although we speculate that the moderate intensity of the resistance training in our study affects the KNDγ-neuron network in the compliant participants mostly via an enhanced gene expression of dynorphin, but to a smaller extent also via the reduced expression of NKB genes.

## Conclusions and implications

In this study, resistance training in postmenopausal women with VMS both affected the GnRH producing neurons, measured as LH levels, and according to the published RTC [12] also the frequency of VMS, probably by means of effects on the thermoregulatory centre in POA in the hypothalamus. The fact that both LH levels and vasomotor symptoms were affected by the resistance training intervention may indicate an effect on the KNDγ-neurons. Further research is needed to better understand the association between VMS, physical activity, the KNDγ neuron network activity and endogenous opioids. By all means increased physical activity could be recommended women to reduce vasomotor symptoms simultaneous with numerous other health effects.

## Supporting information

S1 Checklist CONSORT 2010 checklist of information to include when reporting a randomised trial*. (DOC)

S1 File (DOC)

S1 Data (XLSX)
