# Supplementary material for: Resistance training reduced luteinising hormone levels in postmenopausal women in a substudy of a randomised controlled clinical trial: A clue to how resistance training reduced vasomotor symptoms
Source: PLoS One. 2022 May 26;17(5):e0267613. doi: 10.1371/journal.pone.0267613 (PMC9135255; doi:10.1371/journal.pone.0267613)
Supplement: S1 File — (DOC) [file pone.0267613.s002.doc]

*Beslutad 2013-03-04*

**ANSÖKAN OM ETIKPRÖVNING AV FORSKNING SOM AVSER MÄNNISKOR**

# Information till ansökan, *se Vägledning till ansökan (*[*www.epn.se*](http://www.epn.se/)*)*

**Beroende på vilken forskning som ansökan gäller kommer de uppgifter som efterfrågas att ha olika relevans. Vid ändring av tidigare godkänd ansökan, se *Vägledning till ansökan*.**

**Till Regionala etikprövningsnämnden i:** Linköping

Den regionala etikprövningsnämnd till vars upptagningsområde forskningshuvudmannen hör, se respektive nämnd *(*[*www.epn.se*](http://www.epn.se/)*)*.

Avgift inbetald datum:

Observera att en ansökan aldrig är komplett och därmed kan behandlas förrän blanketten är korrekt ifylld och avgiften är betald.

**Projekttitel:** Effekter av styrketräning på kvinnor med övergångsbesvär

Ange en beskrivande titel på svenska för lekmän. Titeln ska ej innehålla sekretesskyddad information. Ange också i förekommande fall, t.ex. vid klinisk läkemedelsprövning, projektets identitet, forskningsplanens/protokollets nummer, version, datum. Vid ändring av tidigare godkänd ansökan, se [Vägledning till ansökan](http://www.epn.se/media/45470/v_gledning_till_ans_kan_2012-03-27.doc).

Projektnummer/identitet: 20130619 Version nummer: 1 EudraCT nr (vid läkemedelsprövning):

## **Uppgifter som fylls i av den regionala etikprövningsnämnden**

Ansökan komplett: Dnr:

Begäran om ytterligare information (i sak): Begärd information inkommen:

Beslutsdatum: Expeditionsdatum:

**Ansökan avser (gäller även vid b****egäran om rådgivande yttrande):**

Forskning där endast en forskningshuvudman deltar (5 000 kr)

Forskning där mer än en huvudman deltar (16 000 kr)

Forskning där mer än en forskningshuvudman deltar, men där samtliga

forskningspersoner eller forskningsobjekt har ett omedelbart

samband med endast en av forskningshuvudmännen (5 000 kr)

Endast behandling av personuppgifter (5 000 kr)

(När enbart redan befintliga personregister ska användas, t. ex. nationella databaser)

Forskning som gäller klinisk läkemedelsprövning (16 000 kr)

Ändring av tidigare godkänd ansökan enligt 4 § förordning (2003:615) om

etikprövning av forskning som avser människor (2 000 kr)

Om nämnden finner att forskningsprojektet inte faller inom etikprövninglagens tillämpningsområde

önskas ett rådgivande yttrande. (Se [4a och 4b §§ i förordning 2003:615](http://www.epn.se/media/8604/2003_615_4ab.doc) och [Vägledning till ansökan](http://www.epn.se/media/45470/v_gledning_till_ans_kan_2012-03-27.doc))

Ja:  Nej:

**1. Information om forskningshuvudman m.m.**

**1:1 Forskningshuvudman** ([Se p. 1:1 i Vägledning till ansökan](http://www.epn.se/media/43343/vta_p1_1.doc))

Ansökan om etikprövning av forskning ska göras av forskningshuvudmannen. *Med forskningshuvudman avses en statlig myndighet eller en fysisk eller juridisk person i vars verksamhet forskningen utförs.*

Namn: Landstinget i Östergötland

Adress: 58185 Linköping

**1:2 Behörig företrädare för forskningshuvudmannen**

Behörig företrädare är t.ex. prefekt, enhetschef, verksamhetschef. Forskningshuvudmännen bestämmer själva, genom interna arbets- och delegationsordningar eller genom fullmakt, vem som är behörig att företräda forskningshuvudmannen.

Namn: Ninnie Borendal Wodlin Tjänstetitel: Öl, Med Dr

Adress: Kvinnokliniken US Linköping 58185 Linköping

**1:3 Forskare som är huvudansvarig för genomförandet av projektet (kontaktperson)**(Se [p. 9 bil. nr 10 och p. 1:3 i Vägledning till ansökan](http://www.epn.se/media/43346/v_gledning_till_ans_kan_p1_3.doc))

Observera! Den som är huvudansvarig forskare ansvarar för att andra medverkande som ska genomföra projektet har tillräcklig kompetens (vetenskaplig och klinisk) och vid läkemedelsprövning har tillräcklig kunskap om ”Good Clinical Practice” (GCP). Vid doktorandstudier är som regel handledaren huvudansvarig forskare.

Namn: Anna-Clara Spetz Holm Tjänstetitel: Specialistläkare, Docent

Postadress: Kvinnokliniken US 58285 Linköping

E-postadress: anna-clara.spetz.holm@liu.se

Telefon: 010-1033130

Mobiltelefon: 070-3046309

**1:4 Andra medverkande** ([Se p. 9 bil. nr 1 och p. 1:4 i Vägledning till ansökan](http://www.epn.se/media/43349/v_gledning_till_ans_kan_p1_4.doc))

Övriga deltagande forskningshuvudmän samt forskare ansvariga för att lokalt genomföra projektet (kontaktpersoner) anges här eller i bilaga med namn och adresser (se p. 9 bil. nr 1).

Mats Hammar, överläkare, professor, Kvinnokliniken, Universitetssjukhuset, Linköping

Lotta Lindh Åstrand, leg sjuksköterska, med dr, Kvinnokliniken, US, Linköping

Hanna Lindblom, leg sjukgymnast, med master, IMH/ HU/LiU

Peter Söderkvist, professor, IKE/HU/LiU

**1:5 Redovisa tillgång till nödvändiga resurser under projektets genomförande**

(Se p. 9 bil. nr 9 och [p. 1:5 i Vägledning till ansökan](http://www.epn.se/media/43352/v_gledning_till_ans_kan_p1_5.doc))

Ange vem/vilka som har ansvaret (prefekt, verksamhetschef eller motsvarande) för forskningspersonernas säkerhet vid alla enheter/kliniker där forskningspersoner ska delta. Intyg från dessa ansvariga *ska* bifogas (se p. 9 bil. nr 9). Av intyget ska framgå att erforderliga ekonomiska, strukturella och personella resurser finns tillgängliga för att garantera forskningspersonernas säkerhet.

Verksamhetschef Ninnie Borendal Wodlin

**1:6 Ansökan/anmälan till andra myndigheter i vissa fall**

([se p. 1:6 i Vägledning till ansökan](http://www.epn.se/media/43355/v_gledning_till_ans_kan_p1_6.doc))  **Insänd Datum**

a)Vid klinisk läkemedelsprövning: Läkemedelsverket

b)Vid inrättande av biobank: Socialstyrelsen

c)Vid undersökning omfattande joniserande strålning: Strålskyddskommitté

### 2. Uppgifter om projektet

**2:1 Sammanfattande beskrivning av forskningsprojektet**(Se p. 9 bil. nr 2 och [p. 2:1 i Vägledning till ansökan](http://www.epn.se/media/43358/v_gledning_till_ans_kan_p2_1.doc))

Beskrivningen ska kunna förstås av nämndens samtliga ledamöter. Undvik därför terminologi som kräver specialkunskaper. Ange bakgrund och syfte för studien samt den/de vetenskapliga frågeställning (ar) som man söker svar på. Ange de viktigaste undersökningsvariablerna. Beskriv vilka kunskapsvinster projektet kan förväntas ge och betydelsen av dessa. Ange om det är en registerstudie, uppdragsforskning etc. För fackmän avsedd detaljerad information om forskningsplan/protokoll (program) *ska* bifogas som bilaga (se p. 9 bil. nr 2). För utformning av forskningsplan/protokoll se p. 2:1 i Vägledning till ansökan. Ange när datainsamlingen beräknas vara avslutad. En utförligare beskrivning av studiens genomförande *avsedd för lekmän* kan vid behov bifogas den för fackmän avsedda obligatoriska forskningsplanen.

Bakgrund: Svettningar och värmevallningar (flusher) förekommer hos c:a 75% av alla kvinnor efter menopaus. Dessa kan påverka både nattsömn och arbetsförmåga och därmed välbefinnande och humör. Detta sker i samband med att östrogenbildningen från äggstockarna kraftigt minskar. Länge har östrogenbehandling använts som behandling mot flusher och fram till för 10 år sedan hade nära 50% av svenska kvinnor någon period använt sådan behandling. Ett antal studier har visat på risker med sådan behandling för såväl bröstcancer som hjärt-kärl sjukdom och idag rekommenderas hormonell behandling endast vid svåra besvär och då med lägsta möjliga dos och kortast möjliga tid. Vid en egen undersökning 2010 hade färre än 10% hormonbehandling. Däremot angav uppemot en tredjedel av kvinnorna att de hade behov av hjälp mot besvären. Således finns behov av alternativa behandlingar.

Mekanismerna bakom uppkomsten av flusher är inte känd i detalj, men inbegriper en instabilitet i temperaturregeringen i termostaten belägen i hypothalamus. Vid plösligt ändrad inställning i termostaten reagerar kroppen med ökad svettning och ökat ytligt blodflöde och rodnad av huden. Därmed uppkommer en flush. Temperaturcentrum påverkas av en rad olika ämnen, bl. a. av olika transmittorsubstanser som t.ex. beta-endorfin. Vi har i tidigare studier funnit att flusher är mindre vanliga hos kvinnor som tränar regelbundet och kvinnor som börjar träna har i några studier fått minskade besvär. Vi tror att mekanismen bakom lindrande effekter av träning är att fysiskt arbete engagerande stora muskelgrupper stimulerar den hypotalamiska endorfinbildningen, som i sin tur stabiliserar termostaten. En nylig amerikansk multicenterstudie kunde inte påvisa minskade flusher av fysisk aktivitet, men i denna studie innebar den fysiska aktiviteten mest konditionsträning.

Telomerer är repetitiva sekvenser av DNA som finns i änden av däggdjurskromosomer. Vi vet att längden på dessa minskar med stigande ålder (för varje celldelning) och med många kroniska sjukdomar. Studier har visat att telomerlängden kan påverkas av fysisk aktivitet, då testat i huvudsak i form av konditionsträning/aerobisk träning. Brunt fett i kroppen finns framför allt hos nyfödda och har till huvudsaklig uppgift att producera värme, och därmed fås en ökad förbränning. Nyligen har man funnit brunt fett även hos vuxna människor och ett intresse har vuxit angående detta då en ökad andel brunt fett skulle kunna ha positiva effekter på många kroniska sjukdomar. Vi vet också att andelen brunt fett i kroppen kan påerkas på liknande sätt som telomerer enligt ovan. Irisin är ett nyligen upptäckt myokin bildat av muskelvävnad som kan påverka omvandlingen av gult fett till brunt fett. Vi vill nu undersöka om antal och besvär av flusher, livskvalitet, telomer-längd (blodprov), serumnivåerna av irisin respektive andel brunt fett (mätt med Magnet-kamera undersökning) påverkas av 15 veckors styrketräning.

Syfte och vetenskaplig frågeställning:

Syftet är att fastställa om standardiserad styrketräning under 15 veckor minskar antalet och graden av flusher och därmed förbättrar livskvaliteten hos kvinnor med övergångsbesvär. Syftet är också att undersöka om telomerlängden, serumnivåerna av irisin, mängden brunt fett och muskelstyrka påverkas av interventionen. Det långsiktiga syftet är att utarbeta metoder som kan förbättra välbefinnandet och hälsan hos kvinnor med övergångsbesvär.

Den vetenskapliga frågeställningen är att undersöka om ändringen i antal och intensitet av flusher och livskvalitet är större hos kvinnor som genomgått 15 veckors standardiserad styrketräning än i en obehandlad kontrollgrupp.Vi vill också undersöka om telomerlängd, irisin i serum, mängden brunt fett och muskelstyrka förändras mer efter 15 veckors standardiserad styrketräning än i en obehandlad kontrollgrupp.

Viktigaste undersökningsvariablerna:

Primära undersökningsvariabler: Förändring av antal flusher/dygn mätt efter 15 veckors styrketräning registrerad i dagbok

Sekundära undersökningsvariabler: Förändring av svårighetsgraden av flusher mätt efter 15 veckors styrketräning registrerad i dagbok

Förändring i livskvalitet mätt med Women's Health Questionnaire (WHQ; ”sjukdomsspecifikt formulär”) och Shor Form Health Survey 36 (SF-36)

Förändringen av muskelstyrka i arm- och benmuskler mätt genom 8RM (maximal belastning som kan upprepas åtta gånger) vid baseline och efter 3 och 15 v intervention

Andel brunt och vitt fett (MR-kamera)

Irisin mätt i serum

Telomerlängd mätt i blod

Grad av fysisk aktivitet för att kontrollera följsamhet: Träningsdagbok (Friskis & Svettis, bilaga ), IPAQ-skala

Kunskapsvinster och betydelse: Det finns ett stort behov av alternativa behandlingsmetoder för flusher. Teorin bakom flushers uppkomstmekanismer är ej känd i detalj, men vi vet enligt ovan att man vid östrogenbortfallet i klimakteriet får en instabil termostat i hypotalamus. Vi vet också att bl.a. beta-endorfiner påverkar temperaturcentrum. Stöd för denna teori skulle vi få om vi kan visa att träning, som ökar beta-endorfinbildningen har effekt på flusher. Träning har därutöver många positiva effekter på hälsa, sömn och välmående och utgör i så fall ett utmärkt behandlingsalternativ.

Kunskaperna om effekter av styrketräning på telomerer, irisin och brunt fett är ännu begränsade. Vi anser det därför intressant att undersöka om/hur 15 veckors standardiserad styrketräning påverkar dessa variabler hos denna grupp av kvinnor. Vi kommer också att kunna bidra till en validering av en metod där man med MR-kamera mäter brunt fett och se om detta påverkas av interventionen.

**2:2 Vilken/vilka vetenskaplig (a) frågeställning (ar) ligger till grund för projektets utformning?**

Om projektet kan karakteriseras som en hypotesprövning, ange den primära och eventuellt sekundära hypotesen. Hänvisning till mer detaljerad information för fackmän kan ske till bifogad forskningsplan/protokoll enligt punkt 2:1.

Hypotesen är att standardiserad styrketräning under 15 veckor minskar antalet och graden av flusher och därmed förbättrar livskvaliteten hos kvinnor med övergångsbesvär jämfört med en obehandlad kontrollgrupp. Hypotesen är också att telomerlängden, serumnivåerna av irisin och mängden brunt fett ökar av interventionen.

Den vetenskapliga frågeställningen är om ändringen i antal och intensitet i flusher och livskvalitet är större hos kvinnor som genomgått 15 veckors standardiserad styrketräning än i en obehandlad kontrollgrupp. Vi vill också undersöka om telomerlängd, serumnivåerna av gonadotropiner, irisin, mängden brunt fett och muskelstyrka förändras av 15 veckors standardiserad styrketräning jämfört med en obehandlad kontrollgrupp.

**2:3 Redogör för resultat från relevanta djurförsök (Gäller klinisk behandlingsforskning)**

Om djurförsök inte utförts ange skälen till detta.

Inte tillämpbart i detta fall då vi inte kan få djur att träna styrketräning under de premisser vi önskar.

**2:4 Redogör översiktligt för undersökningsprocedur, datainsamling och datas karaktär** ([Se p. 9 bil. nr 5 och p. 2:4 i Vägledning till ansökan](http://www.epn.se/media/43361/v_gledning_till_ans_kan_p2_4.doc))

Av beskrivningen ska framgå hur projektet planeras genomföras. Beskriv insamlade datas karaktär. Ange hur datas tillförlitlighet säkerställs (t.ex. kvalitetskontroll/monitorering). Vid enkäter och intervjuer ska beskrivas tillvägagångssätt och t.ex. frågors innehåll och hur slutsatser dras. Enkäter och skattningsskalor *ska* bifogas (se p. 9 bil. nr 5). För medicinsk forskning ska anges t.ex. typer av ingrepp, mätmetoder, antal besök, tidsåtgång vid varje försök, doser och administrationssätt för eventuella läkemedel och/eller isotoper, blodprovsmängd (även ackumulerad mängd vid multipla försök). Ange om och på vilket sätt undersökningsprocedur m.m. skiljer sig från klinisk rutin. Om en behandling studeras för första gången på människa ska detta framgå och relevanta säkerhetsrutiner beskrivas. Ange proceduren för att ge den eventuella behandling efter projektets slut, som kan erfordras. Ange procedur för insamling av biologiskt material. Redogör för datakällor och procedurer vid behandling av personuppgifter. För mer detaljerad information kan hänvisning ske till bilagd forskningsplan.

Studien genomförs som en öppen, prospektiv, randomiserad kontrollerad interventionsstudie.

Efter skriftligt och muntligt informerat samtycke från forksningspersonerna och inklusion i studien kommer dessa att randomiseras till antingen interventionsgrupp (IG) eller kontrollgrupp (KG). Randomiseringslista tas fram av oberoende person genom datagenererat program. Fördelningen mellan grupperna kommer att vara 1:1. Randomisering kommer att ske i konsekutiv ordning med hjälp av slutna kuvert.

Baslinjedata båda grupperna: Fysikalisk undersökning (auskultation av hjärta/lungor) blodtryck, vikt och längd, Body Mass Index (BMI), bukomfång, blodprover (Hb, LH och FSH).

Interventionsgruppen: Standardiserad styrketräning som leds av erfaren sjukgymnast och ledare under 15 veckors tid i lokaler hos Friskis&Svettis, varav de tre första veckorna med lägre belastning. Varje styrketräningstillfälle beräknas ta 45-60 minuter inklusive uppvärmning och stretching. Ledarledd träning erbjuds 3 gånger i veckan, varav ett tillfälle är obligatoriskt för avstämning av träningsprogrammet. Inför träningsstart görs baslinje styrketest enligt 8RM. Vid deltagarens första träningstillfälle närvarar endast sjukgymnast och aktuell deltagare så att det finns gott om tid och möjlighet att prova ut rätt belastning och inställning av alla maskiner samt att öva in en korrekt teknik i alla övningar. Vid följande träningstillfällen deltar flera deltagare samtidigt, men alla kan få råd och stöttning av närvarande sjukgymnast. Efter 3 veckor och efter träningsperiodens slut görs ett nytt individuellt styrketest enligt 8 RM. Forskningspersonerna får förutom 3 styrketräningspass per vecka komplettera sin träning med ytterligare träningstillfällen i form av "vardagsmotion". För program och mer detaljer se forskningsprogram.

Kontrollgruppen: Får information att inte förändra sin grad av fysisk aktivitet under studieperioden. Efter avslutat deltagande i studien (15 v) erbjuds kontrollgruppen en vägledning om fysisk aktivitet enligt YFA och en ledarledd gyminstruktion samt 3 månaders gym-kort på Friskis&Svettis.

Följande mätningar kommer att göras i både grupperna (IG + KG):

- Flusher: Mäts med dagbok angående frekvens och svårighetsgrad av flusher (se bilaga). Dagbok förs 2v som screening, därefter 3:e; 7:e, 11:e och 15:e veckan samt en vecka 6 respektive 24 månader efter avslutad intervention/respektive medverkan i kontrollgrupp. Påminnelse kommer at skickas till kvinnnan via SMS eller e-post några dagar innan registreringen ska påbörjas.

- Hälsorelaterad Livskvalitet (HRQoL) med WHQ, SF-36 vid baslinjen, efter 15 veckor samt 6 respektive 24 månader efter avslutad intervention/respektive medverkan i kontrollgrupp.

- Mätning av fysisk aktivitetsgrad: International Physical Activity Questionnaires (IPAQ)-skalan vid baslinjen, efter 15 veckor samt 6 respektive 24 månader efter avslutad intervention/respektive medverkan i kontrollgrupp.

- Mängd vitt och brunt fett: MR-kamera vid baslinjen samt efter 15 v, beräknas ta ca 45 minuter/gång.

- Irisin: Blodprov vid baslinjen och efter 15 v.

- Telomerlängd: Blodprov vid baslinjen och efter 15 v

- Vikt och längd (BMI): vid baslinjen samt efter 15 v

- Bukomfång: vid baslinjen samt efter 15 v

- Registrering av ev bieffekter, förändrat hälsotillstånd resp förändrat läkemedelsintag görs fortlöpande under studiens gång av kvinnan själv i dagboken, av testledaren i samband med styrketräningspass samt i Case Record Form vid besök på Kvinnokliniken.

- Besök på Kvinnoklinkens mottagning: Ett besök för screening (se baslinjedata ovan), ett besök för randomisering (och blodprovstagning för telomerer och irisin) och ett besök efter 15 veckor. Dessa beräknas ta ca 1 timme i anspråk per tillfälle.

- Uppföljning 6 respektive 24 månader efter avslutad intervention/respektive medverkan i kontrollgrupp.

Tester enbart för IG:

- Muskelstyrka: Muskelstyrka utvärderas genom test av 8RM i olika övningar vid baslinjen, efter 3 veckor samt efter 15 veckor, se forskningsprogram

Källdata är Informerat samtycke, dagböcker, besvarade formulär, CRF, patientens journal, labsvar för studiespecifika prover. Källdata kommer att sparas under 10 år efter avslutad studie och arkiveras i enlighet med regler i Landstinget i Östergötland.

**2:5 Redogör för om insamlat biologiskt material kommer att förvaras i en biobank**

([Se p. 2:5 i Vägledning till ansökan](http://www.epn.se/media/43364/v_gledning_till_ans_kan_p2_5.doc))

*Med biobank avses biologiskt material från en eller flera människor som samlas och bevaras tills vidare eller för en bestämd tid och vars ursprung kan härledas till den eller de människor från vilka materialet härrör.*Redogör för var och hur prover som ska sparas förvaras, kodningsprocedurer och villkor för utlämnande av prover. Ange huvudman för biobanken.

Blodprov för bestämning av telomerlängd och bestämning av irisin kommer att sparas i provsamling inom befintlig biobank vid Kvinnokliniken i Linköping, LiÖ för att analyseras efter studiens slut. Insamlat biologiskt material kommer att förvaras enligt biobankens riktlinjer och villkor för utlämnande kommer att regleras i biobanksavtal/föreskrift. Blodprover kommer att kodas med patienspecifik studiekod, initialer och datum för provtagning.

**2:6 Dokumentation, dataskydd och arkivering** ([Se p. 2:6 i Vägledning till ansökan](http://www.epn.se/media/43367/v_gledning_till_ans_kan_p2_6.doc))

Redogör för hur undersökningsprocedurer och eventuella ingrepp dokumenteras. Ange om band- och videoinspelningar används. Om materialet ska kodas, ange proceduren, vem som förvarar kodlistor/kodnycklar och vem eller vilka som har tillgång till dem, var och hur länge de förvaras samt om materialet kommer att anonymiseras eller förstöras. Redogör för vilken tillgänglighet datamaterialet har och hur det förvaras samt hur erforderligt sekretesskydd erhålls.

Källdata är samtycken, dagböcker, besvarade formulär, CRF, patientens journal, labsvar för studiespecifika prover. Källdata kommer att sparas under 10 år efter avslutad studie och arkiveras i enlighet med regler i Landstinget i Östergötland. Studiespecifika formulär kommer att vara kodade och patientidenifikationslista förvaras så att utomstående inte kan få tillgång till denna. Enbart personer knutna till forskningsprojektet kommer att ha tillgång till patientidenifikationslistan och andra källdata (förutom journalen). Sedvanlig patientjournal kommer att föras.

**2:7 Redogör för tidigare erfarenheter (egna och/eller andras) av den använda
proceduren, tekniken eller behandlingen**

Särskilt angeläget är att redovisning av risker för komplikationer görs tydliga och i förekommande fall med angivande av relevanta publikationer. Vid nya behandlingar av patienter, t.ex. med läkemedel, bör anges hur många patienter (med aktuell eller annan åkomma) som tidigare erhållit föreslagen behandling, läkemedelsdosering (eller annan dosering) samt hur långa behandlingsperioder som studerats.

Styrketräningen är standardiserad och ledd av erfaren ledare som är sjukgymnast. Träningen är anpassad för kvinnor i aktuell ålder. Det kommer att ske en succesiv belastningsökning under studiens gång i takt med att studiepersonerna blir starkare, denna ökning kommer att anpassas individuellt.

Säkerhet; Interventionsgruppen: Under interventionens gång tillfrågas kvinnorna varje vecka i samband med ledarledd träff om några problem såsom skador uppstått eller om andra hälsoproblem finns. Detta registreras i särskilt formulär av träningsledaren och av forskningspersonen själv i träningsdagboken.

Båda grupperna: I flushdagbok kan deltagaren själv registrera hälsoproblem som inträffar under studien. Alla kvinnor kommer att tillfrågas om tillkommande sjukdomar och läkemedel i samband med avslutande besök efter 15 veckor samt efter 6 respektive 24 månader.

**3. Uppgifter om forskningspersoner**

**3:1 Hur görs urvalet av forskningspersoner?** ([Se p. 9 bil. nr 3 och p. 3:1 i Vägledning till ansökan](http://www.epn.se/media/43370/v_gledning_till_ans_kan_p3_1.doc))

*Med forskningsperson avses en levande människa som forskningen avser.* Ange urvalskriterier (inklusion och exklusion). Redogör för på vilket sätt forskaren kommer i kontakt med/får kännedom om lämpliga forskningspersoner. Om annonsering sker, *ska* annonsmaterialet insändas som bilaga (se p. 9 bil. nr 3). Om t.ex. barn eller personer som tillfälligt eller permanent inte är kapabla att ge ett eget informerat samtycke ska ingå i projektet, ska detta särskilt motiveras. Om vissa grupper utesluts från deltagande i projektet ska detta särskilt motiveras.

Inklusionskriterier:

•Postmenopausala kvinnor med minst 4 medelsvåra eller svåra flusher per dygn eller 28 per vecka

Med postmenopausala kvinnor menas;

- ≥12 månader sedan senaste menstruationsblödningen

- hysterektomerade kvinnor med FSH >20mIU/mL

- kvinnor med Mirenaspiral med FSH >20mIU/mL

- inducerad menopaus (pga. ooforektomi)

- inducerad menopaus (pga. ooforektomi eller efter avslutad kemoterapi eller strålning då med minst 2 års amenorré)

• Ålder ≥ 45 år

• God hälsa och fysiska möjligheter att delta i organiserad styrketräning enligt beskrivning under metoddelen och annan träning tre gånger per vecka om 60 min per pass

• Behärskar svenska språket i tal och skrift

•Lämnat sitt samtycke till att delta i studien

Exklusionskriterier:

• Fysisk aktivitet > 75 minuter/vecka (kan delas upp i flera kortare perioder på minst 10 minuter) av medel till hård belastning, dvs. upplevs arbetssam och leder till att personen andas kraftigare än normalt (tex. rask promenad, stavgång, cykling, gymnastik, löpning eller annan form av motionsaktivitet)

• Fysisk aktivitet >150 minuter/vecka av låg belastning, ex. promenad i lugnt tempo, förflyttning till och från arbete ex. cykel.

• Hb värde < 110g/l.

• Blodtrycksförhöjning >160 systoliskt och/eller >100 mmHg diastoliskt

• Hormonbehandling (HT) för klimakteriebesvär de senaste 2 månaderna (lokal behandling för slemhinnebesvär är tillåten respektive peroral östriolbehandling)

• Användning av SSRI, SNRI, eller annan behandling (inkl naturläkemedel) med möjlig effekt på övergångsbesvär (kan delta om behandlingen och flusher inte bedöms ha någon relation tidsmässigt och behandlingen är stabil)

• Outredd/instabil sjukdom som kan tänkas påverka förekomst av flusher som t.ex. tyroideasjukdom

• Annat medicinskt tillstånd som av läkare bedöms vara olämpligt att förena med fysisk aktivitet eller deltagande i studien

Forskaren kommer i kontakt med forskningspersonerna genom lokala anslag och annonsering i lokal press (annons bifogas).

**3:2 Ange relationen mellan forskare/försöksledare och forskningspersonerna**

Behandlare (t.ex. läkare, psykolog, sjukgymnast) - forskningsperson (t.ex. patient, klient)

Kursgivare (lärare) - student

Arbetsgivare - anställd

Annan relation som kan tänkas medföra risk för påverkan. Beskriv:

3:3 Redogör för det statistiska underlaget för studiepopulationens (-ernas)/ undersökningsmaterialets (-ens) storlek [**(Se p. 3:3 i Vägledning till ansökan)**](http://www.epn.se/media/43373/v_gledning_till_ans_kan_p3_3.doc)

Redovisa statistisk styrka, så kallad ”power”- beräkning eller redovisa motsvarande överväganden som tydliggör studiens möjligheter att besvara frågeställningarna.

Vi planerar att starta studien som en pilotstudie omfattande 20 kvinnor, där 10 kvinnor ska ingå i interventionsgruppen och 10 i kontrollgruppen. Efter detta gör vi en urvalsstorleksberäkning för att dimensionera den fullskaliga studien.

Erfarenheter från tidigare studier av kvinnor i liknande ålder och med behandlingar mot flusher har visat att ca 30 kvinnor i interventions- respektive kontrollgruppen (som fullföljer hela forskningsprogrammet och där skillnaden i ändring är ungefär 30% från utgångsnivån) bör vara tillräckligt för att med 80% power kunna visa en signifikant effekt p< 0,05.

**3:4 Kan forskningspersonerna komma att inkluderas i flera studier samtidigt
eller i nära anslutning till denna? I så fall, vilken typ av forskning?**

([Se p. 3:4 i Vägledning till ansökan](http://www.epn.se/media/43376/v_gledning_till_ans_kan_p3_4.doc))

Nej.

3:5 Vilket försäkringsskydd finns för de forskningspersoner som deltar i projektet?

Det åligger forskningshuvudmannen att kontrollera att det finns försäkring som täcker eventuella skador som kan uppkomma i samband med forskningen.

Patientskadelagen och Patientförsäkringen. Interventionsgruppen omfattas även under interventionen av Friskis&Svettis olycksfallsförsäkring omfattande tid i Friskis&Svettis lokaler och resa till/från träningen.

**3:6 Vilken ekonomisk ersättning eller andra förmåner utgår till de forskningspersoner
som deltar i projektet och när betalas ersättningen ut?** Utförligare beskrivning kan lämnas i bilaga. ([Se p. 9 bil. nr 11 och p. 3:6 i Vägledning till ansökan](http://www.epn.se/media/43379/v_gledning_till_ans_kan_p3_6.doc))

Ersättning för obehag och besvär. Ange belopp (före skatt):

Ersättning för förlorad arbetsinkomst  Ja  Nej

Reseersättning  Ja  Nej

Befrielse från kostnader för läkemedel  Ja  Nej

Befrielse från andra kostnader. Vilka?

Andra förmåner. Vilka? Träningskort på F&S värde ca 700 kr

När betalas ersättningen ut? För IG vid randomisering, för KG efter 15 v deltagande i kontrollgrupp är slutförd

Ingen ersättning betalas ut

### 4. Information och samtycke

4:1 Proceduren för och innehållet i den *information* som lämnas då forskningspersoner tillfrågas om deltagande

(Se p. 9 bil. nr 4 och [**Vägledning till forskningspersonsinformation**](http://www.epn.se/media/45467/v_gledning_till_forskningspersonsinformation_2012-03-27.doc).)

Enligt 16 § lag (2003:460) om etikprövning av forskning som avser människor ska forskningspersonen informeras om den övergripande planen för forskningen, syftet med forskningen, de metoder som kommer att användas, de följder och risker som forskningen kan medföra, vem som är forskningshuvudman, att deltagande i forskningen är frivilligt och forskningspersonernas rätt att när som helst avbryta sin medverkan. Beskriv hur och när information ges och vad den innehåller. Ange vem som informerar. Normalt ska en kortfattad och lättförståelig skriftlig information ges. Denna skriftliga information *ska* bifogas ansökan (se p. 9 bil. nr 4). Om ingen eller ofullständig information ges, måste skälen för detta noggrant anges.

Patienterna kommer att rekryteras genom annonsering i media (annons bifogas) samt lokala anslag.

Patientinformation sändes till personer som anmäler intresse av att delta i studien via telefon, e-post eller brev. I samband med telefonsamtal sker en snabb avstämning avseende inklusions- och exklusionskriterier och för de kvinnor som verkar lämpliga bokas ett screeningbesök där muntlig information ges av sjuksköterska och läkare involverade i studien och möjlighet att ställa frågor finns. Därefter kan forskningspersonen ge sitt muntliga och skriftliga informerade samtycke. Forskningspersonen informeras om PUL och biobankslagen samt rätten att erhålla registerutdrag och att få sina blodprover kasserade.

Den skriftliga informationen upplyser om rätten om att avbryta sitt deltagande när som helst utan att detta påverkar hennes framtida vård och behandling.

**4:2 Hur och från vem inhämtas *samtycke*?** (Se [Vägledning till forskningspersonsinformation](http://www.epn.se/media/45467/v_gledning_till_forskningspersonsinformation_2012-03-27.doc))

Beskriv proceduren; vem som frågar, när detta sker och hur samtycket dokumenteras. Utförlig redovisning är särskilt viktig då barn eller personer med nedsatt beslutskompetens ingår i studien, likaså vid studier av en grupp/grupper, t.ex. skolklasser, föreningar, organisationer, företag, kyrkosamfund, församlingar eller grupper som interagerar inom sociala medier.

Läkare kommer att informera patienten och inhämta samtycke i samband med första besöket i studien. Enligt ovan ger forskningspersonen sitt muntliga och skriftliga informerade samtycke, vilket dokumenteras i patientjournalen. Forskningspersonen informeras om PUL och biobankslagen samt rätten att erhålla registerutdrag och att få sina blodprover kasserade. En patientidentifikationslista kommer att upprättas och anmälan om behandling av känsliga personuppgifter kommer att lämnas till Personuppgiftsansvarig vid Landstinget i Östergötland.

### 5. Forskningsetiska överväganden

5:1 Redogör för alla risker som deltagandet kan medföra

Dessa kan vara t.ex. fysisk eller psykisk skada, smärta, obehag eller integritetsintrång på kort eller lång sikt. Ange vilka åtgärder som har vidtagits för att förebygga riskerna som nämns ovan samt vilken beredskap som finns för att hantera sådana komplikationer. Ange vilka/de metoder som kommer att användas för att efterforska, registrera och rapportera oönskade händelser.

Risken med interventionen är skador relaterade till träning, fr a överbelastningsskador. Risken minimeras m h a en infasningsperiod som genomförs med lägre belastning och regelbunden kontakt och ”övervakning” av erfaren sjukgymnast/träningsledare.

Risken med blodprovstagningen är minimal och inskränker sig till obehag vid stick och lokalt hematom.

MR-undersökning kan av vissa personer upplevas obehaglig då undersökningen genomförs i utrustning med trånga utrymmen. Undersökningen kommer att genomföras efter information till forskningspersonen och av personal med stor erfarenhet av dessa undersökningar.

Kvinnor som kan inkluderas i studien ska ha besvärande värmevallningar och svettningar vilket kan påverka livskvalitet och sömn. De kvinnor som randomiseras till kontrollgruppen kommer att vara utan behandling under 15 veckor. Kvinnan informeras noggrant innan samtycke inhämtas och känner till förutsättningarna för studien. Kvinnan har också full rätt att avbryta sitt deltagande och då få nödvändig behandling för sina klimakteriebesvär.

Integriteten påverkas alltid men forskningspersonens autonomi hotas inte då varje deltagare själv kan välja att avstå delar av studien eller helt avbryta sitt deltagande utan att ange orsak.

5:2 Redogör för möjlig nytta för de forskningspersoner som ingår i projektet (gäller särskilt behandlingsforskning)

Fördelar för individen är de hälsobefrämjande effekterna av träning och erhållen hälsokontroll. Fördelar för populationen är att resultaten kan komma att leda till evidensbaserade råd om möjlig självhjälp vid övergångsbesvär.

**5:3 Identifiera och precisera om eventuella etiska problem (fördelar/nackdelar) kan uppstå i ett vidare perspektiv genom projektet** (Se [p. 5:3 i Vägledning till ansökan](http://www.epn.se/media/43382/v_gledning_till_ans_kan_p5_3.doc))

Här kan redovisas om exempelvis vissa grupper (andra än de forskningspersoner som ingår i forskningsprojektet) kan komma att utpekas/få hjälp som ett resultat av studien.

Helsingforsdeklarationen kommer att följas. Fördelar i ett vidare perspektiv är att forskningspersonerna eventuellt kommer att fortsätta med sin träning och därmed ha fortsatta hälsoeffekter.

### 6. Redovisning av resultaten

**6:1 Hur garanteras forskningshuvudmannen och medverkande forskare tillgång till data (anges vid t.ex. uppdragsforskning) och vem ansvarar för databearbetning och rapportskrivning?**([Se p. 6:1 i Vägledning till ansökan](http://www.epn.se/media/43385/v_gledning_till_ans_kan_p6_1.doc))

För databearbetning, analys och rapportskrivning ansvarar samtliga medarbetare i studien, dvs Anna-Clara Spetz Holm, Lotta Lindh-Åstrand, Hanna Lindblom, Peter Söderkvist och Mats Hammar.

6:2 Hur kommer resultaten att göras offentligt tillgängliga? Kommer studien att insändas för publicering i tidskrift eller publiceras på annat sätt? ([**Se p. 6:2 i Vägledning till ansökan**](http://www.epn.se/media/43388/v_gledning_till_ans_kan_p6_2.doc))

Ange i vilken form resultaten planeras offentliggöras samt tidsplan för detta.

Resultaten avses publiceras i internationella refereegranskade tidskrifter och vid internationella och nationella konferenser samt kommer att registreras i offentlig databas.

6:3 På vilket sätt garanteras forskningspersonernas rätt till integritet när materialet offentliggörs/publiceras?

Redovisas resultat på statistisk gruppnivå? Beskriv procedurer eller metoder för avidentifiering/anonymisering.

Resultatredovisningen kommer att göras på sådant sätt att inte enskilda individers identitet kan röjas. Resultaten kommer att redovisas på gruppnivå; kontrollgrupp vs interventionsgrupp.

7. Redovisning av ekonomiska förhållanden och beroendeförhållanden

Redovisning enligt punkterna 7:1-7:3 syftar till att tydliggöra alla direkta eller indirekta förhållanden, som kan tänkas påverka forskarens relation till forskningspersonerna (vid t.ex. informations-, samtyckes-, genomförandeprocedurer).

7:1 Vid uppdragsforskning

Ange uppdragsgivaren t.ex. ett företag (vid klinisk läkemedelsprövning eller prövning av andra nya produkter), en organisation eller en myndighet.

Namn:       Kontaktperson:

Adress:       Telefon/mobiltelefon:

Ange uppdragsgivarens relation till forskningshuvudmannen/medverkande forskare, t.ex.
anställningsförhållande

**7:2 Redovisa eventuella ekonomiska överenskommelser med uppdragsgivare eller**

**andra finansiärer (namn, belopp)**

Vid klinisk läkemedelsprövning bör hänvisning ske till ingånget avtal med sjukvårdshuvudmannen. Liknande överenskommelser kan förekomma vid annan uppdragsforskning och bör redovisas på samma sätt. Separata överenskommelser med den/de som ska genomföra forskningen ska redovisas. Belopp som kommer att erhållas för studien/ersättning till kliniken/genomföraren, vad ersättningen bör täcka och ev. belopp som erhålls per forskningsperson, bör också anges här.

Inga, studien finansieras av forskningsmedel från berörda forskare

7:3 Redovisa forskningshuvudmannens, huvudansvarig forskares och medverkande forskares egna intressen

Här redovisas t.ex. aktieinnehav, anställning, konsultuppdrag i finansierande företag, eget företag som kan få (direkt eller indirekt) ekonomisk vinst av forskningen.

Finns inga sådana aspekter.

**8. Undertecknande**

Behörig företrädare för sökande forskningshuvudman enligt p. 1:2.

Ort: Linköping Datum: 20130619

Signatur: __________________________________________________________________

Namnförtydligande: Ninnie Borendal Wodlin

Tjänstetitel: Verksamhetschef

Undertecknad forskare som genomför projektet (kontaktperson) enligt p. 1:3 intygar härmed att forskningen kommer att genomföras i enlighet med ansökan.

Ort: Linköping Datum: 20130619

Signatur: _________________________________________________________________

Namnförtydligande: Anna-Clara Spetz-Holm

Tjänstetitel: Docent, specialistläkare

**9. Förteckning över bilagor** ([Se p. 9 i Vägledning till ansökan](http://www.epn.se/media/43391/v_gledning_till_ans_kan_p9.doc))

Dokument som, i tillämpliga fall, ska bifogas *om inte motsvarande information finns i blanketten* har markerats med x. Markera de bilagor som skickas in med denna ansökan.

| **Insänd med ansökan** | **Bil nr** | Beskrivning | **Klinisk läkemedels-**  **prövning** | **Annan forskning** |
| --- | --- | --- | --- | --- |
|  | 1 | Deltagande forskningshuvudmän och medverkande forskare (kontaktpersoner) vid forskning där mer än en forskningshuvudman deltar. Se p. 1:4 | x | x |
|  | 2 | För fackmän avsedd forskningsplan, vid behov även för lekmän avsedd bilaga. Se p. 2:1 och Vägledning till forskningsplan/forskningsprotokoll (program) | x | x |
|  | 3 | Annonsmaterial för rekrytering av forskningspersoner. Se p. 3:1 och i Vägledning till ansökan p. 3:1 | x | x |
|  | 4 | Skriftlig information till dem som tillfrågas. Se p. 4:1 och Vägledning till forskningspersonsinformation och (i förekommande fall) separat samtyckesformulär | x | x |
|  | 5 | Enkät, frågeformulär. Se p. 2:4 | x | x |
|  | 6 | Gemensam EU blankett (gäller fr.o.m. den 1 maj 2004), gäller även vid ändring. För information se Läkemedelsverkets hemsida, [www.lakemedelsverket.se](http://www.lakemedelsverket.se/) | x |  |
|  | 7 | Sammanfattning av protokollet på svenska | x |  |
|  | 8 | Prövarhandbok alt. bipacksedel/produktresumé/IB | x |  |
|  | 9 | Intyg från verksamhetschef/motsv. om resurser för forskningspersonernas säkerhet. Se p. 1:5 och förslag till utformning av resursintyg i Vägledning till ansökan p. 1:5 | x | x |
|  | 10 | CV för forskare (samma som p. 1:3) med huvudansvar för genomförandet, redovisa forskarens (- arnas) kompetens av relevans för studien. Se Vägledning till ansökan p. 1:3 | x | x |
|  | 11 | Beskrivning av ersättning till forskningspersoner. Se p. 3:6 och  i Vägledning till ansökan p. 3:6 | x | x |

**Övriga bilagor som bifogas ansökan:**

12. Bilagor patientinfo till de kvinnor som kan tänka sig genomgå MR undersökning

Summary in English for PLOS ONE:

Since application to the Ethical Committee has to be written in Swedish we here summarize in English what is included in the application:

The project was called; Effects of Resistance Training on women with climcateric symptoms mainly vasomtor symptoms. The application was signed by the head of the department of Obsterics and Gynaecology Ninnie Borendahl Wodlin June 2013 and she also certified that sufficient resources existed at the department to perform the study. The study was planned to be performed at one single unit, the Department of Obsterics and Gynecology, University Hospital of Linköping, being a part om the main Health Care provider The County Council of Östergötland, Sweden. Responsible applicant was associate professor Anna-Clara Spetz Holm.

Point 2 is a summary of the planned projet with background regarding menopause, the possible mechanisms behind vasomotor symptoms, Quality of Life, theories about telomere length, browning of white fat, inflammation.

The scientific questions are summerized on page 5 - 6, including several questions not relevant to the present paper submitted to PLOS ONE. The main outcome was changes in hot flushes in the group on 15 weeks of resistance training with a number of other planned outcomes regading changes in biomarkers measured in blood (like cytokines, markers of oxidative stress, gonadotropins etc), telomere length, muscle strength, volume of muscle and adipose tissue (separated as white and brown fat) measured with Magnetic Resonance Imaging; MRI).

On pages 7-8 the project is described as an open, controlled, randomized study and how data will be collected, i.e. at a screening visit, a baseline visit (when randomization is performed) and a 15-week visit after intervention or as the last visit in the control group. This includes fasting blood sampling, measurements of anthropemtric data, filling in of questionnaires and planning for MRI. Point 2.5 deals with how Biobank issues are planned according to Swedish Legislation. Point 2.6 deals with documentation (in the individual's medical record, in CRF and how archieving is planned.

Point 2.7 descibes the intervention and how possible risks are avoided and also how adverse events will be taken care of and registered.

Point 3.1 describes the inclusion and exclusion criteria, point 3.2 describes the relationsship between the researcher and the study participant.

Point 3.3 describes a planned pilot study and the sample size calculation which at that point suggested 30 women in the intervention group and 30 in the control group. The pilot study performed later appeared to show that 20 women in each group would suffice.

Point 3.4 stated that the participating women could not be included in another study in parallell with the present study and point 3.5 described the insurance situtation for the participants.

Point 3.6 descibes that no economical incentives were planned but that the women did not have to pay entrance fees to the gym where the resistance treining took place.

Point 4 (pages 12-13) descibed the process of recruiting women through advertising, the information given to the women, how informed consent would be collected both orally and in writing.

Point 5 conserns the ethical considerations with description of risks such as overuse injuries(with risks minimized by a gradual increase of the wheigts, an experienced physiotherapist participating etc), blood sampling, MRI (with separate exclusion criteria), delayed treatment of 15 weeks (for the women randomized to the control group). Here we also emphasized that the participants could withdraw there consent at any time without any motivation to why and with guarantees that they could be given another therapy of their vasomotor symptoms.

Point 5.2 discusses possible advantages for the women (such as general health benefits from engaging in physical exercise and releif from vasomtor symptoms) and for society (with a possible evidence-based alternative therapy of vasomtor symptoms).

Point 5.3 deals with other possible risks or benefits where we state that we will follow the Helsinki Declaration and that a possble long-term effect would be long-term changes of physical avtivity habits.

Points 6.1-6.2 on pages 14-15 deal with how results will be obtained, who will be responsible for the work leading to results and how they will be published (in international peer-reviewed journals). Point 6.3 states that the integrity of the individual woman is not threatened because analyses will only be performed and presented on a group level.

Pont 7 on page 15 states that the researchers do not have any private economical interests in the project and that the external funding does not affect the way that the project is performed and how results are presented or used.

Point 8 includes signatures.

Point 9 summarizes the appendices included in the application such as Project plan, Advertisment planned to be used, information to the participants in the study and a separate information about MRI, questionnaires planned to be used, cetificate from the department head, and CV:S for the participating researchers.

Linköping 2021-11-08

Mats Hammar

Below is the program of the project in English

Program

Health effects of resistance training on postmenopausal women

Principal investigator: Mats Hammar, MD, professor, Linköping University

Collaborators: Anna-Clara Spetz Holm, MD, PhD, Lotta Lindh Åstrand, RN, PhD. Hanna Lindblom, MSc, reg physiotherapist, Heriberto-Rodriguez-Martinez, professor, Marie Rubér, PhD, Peter Söderkvist, professor, Magnus Borga/Olof Dahlqvist Leinhard, Pontus Boström, ass professor, Carina Berterö, RN, Professor.

Purpose and aims:

Today’s women will live more than a third of their lives after menopause, i.e. with a changed hormonal and metabolic state. The purpose of the study is to establish possible beneficial health effects including decreased climacteric symptoms, inflammatory burden, white adipose tissue mass and increased brown adipose tissue from 15 weeks of supervised resistance training (RT) on postmenopausal women. End-points include effects from RT in postmenopausal women on

a/ clinical outcomes (number and severity of vasomotor symptoms, well-being, Health-Related quality of Life (HRQoL), Body Mass Index (BMI), abdominal height, muscle strength and mass, browning of fat),

b/ diagnostic variables (production of myokines as irisin, immunological markers) and

c/ genetic variables (length of telomeres).

Survey of the field:

Today’s women will live more than a third of their lives after menopause. Menopause is often characterized by a series of clinical signs and symptoms including vasomotor symptoms, sleep and mental disturbances, and symptoms from urogenital atrophy. The vasomotor and urogenital symptomatology is predominant, although other signs are seen such as osteoporosis and loss of muscle mass, replaced by accumulation of white fat, as well as inflammatory changes in the fat tissue, with immunologic and metabolic dysfunctions as consequences.

Vasomotor symptoms: Vasomotor symptoms like hot flushes and sweating are reported by about 75% of all women around menopause and may impair well-being, mental state, daily activities, including professional work, and night sleep. The symptoms usually persist for years (median 5 years) but are sometimes life-long. In women with breast cancer vasomotor symptoms are even more common than in women in general. Also men with prostatic cancer treated with castration therapy suffer from these symptoms and to the same extent as menopausal women.

Treatment of vasomotor symptoms: Until about ten years ago almost every other middle-aged woman in the Western world including Sweden used hormone therapy (HT) with combined estrogens and synthetic progesterone (progestagens). HT effectively diminishes vasomotor symptoms (Shen and Stearns 2009) according to a Cochrane meta-analysis by about 75% compared to placebo (Maclennan 2004). Since HT, however, has been shown to increase the risk of cardiovascular and thrombo-embolic disease, as well as breast cancer, general recommendations are to use HT only in severe cases with lowest possible dosis for the shortest time possible (Rossouw 2002). This has led to decreased use of HT and in a previous study in 2010 we found that less than 10 % of women in Östergötland, Sweden used HT. On the other hand about every third woman stated that she had symptoms that she wanted to be cured from. There is thus a clear need for alternative treatment.

Some pharmacologic alternatives exist, e.g. antidepressants, but none is by far as effective as HT (Loprinzi CL et al 2002, Albertazzi 2007, Shen and Stearns 2009). Acupuncture has in some studies been shown to reduce vasomotor symptoms, as suggested by inducing production of endorphins, but not as effectively as HT (Wyon 1995, Nedstrand 2005, Cohen 2003, Nir 2007, Wyon 2004). Also behavioral therapy has been found to induce some reduction of the symptoms (Nedstrand et al 2005).

Mechanisms behind the vasomotor symptoms: Vasomotor symptoms are elicited from the thermoregulatory centre in the hypothalamus, which usually regulates body temperature in a stable way. If stability of the thermoregulatory centre is lost it may suddenly decrease its set point which leads to reactions that decrease central body temperature (Hammar et al 1990; Wyon et al 1995). As a reaction cutaneous blood vessels dilate, which leads to increased skin temperature and energy loss by irradiation of heat, and activation of sweat glands which causes energy loss by means of evaporation.

Sex steroid levels (estrogens and androgens) in the hypothalamus probably affect the stability of the thermoregulation by stimulating the production of hypothalamic β-endorphins. Nappi and coworkers (1990) found higher levels of ß-endorphins in cerebrospinal fluid from women of fertile age than from postmenopausal women. After menopause or castration the sex steroids decrease leading to a fall in hypothalamic β-endorphins and a less stable thermoregulation with sudden drops in the set point and vasomotor symptoms.

Exercise and vasomotor symptoms: Already in the early 1990:ies (as the first group worldwide) we reported that vasomotor symptoms were less prevalent in women who participated in regular physical exercise (Hammar et al 1990) which we confirmed in a later cross-sectional study (Ivarsson et al 1998). Women who were randomized to, and adhered to the intervention with regular exercise reported decreased vasomotor symptoms and increased quality of life (Lindh-Åstrand et al, 2004). According to a Cochrane review it is still, however, uncertain if physical exercise reduces vasomotor symptoms. The scientific data are scarce and exercise has been poorly defined with a mixture of aerobic and resistance training in the available studies. Another problem with the previous studies is insufficient compliance to the treatment (Daley 2011).

Mechanisms behind the effects of exercise on vasomotor symptoms: One theory to why physical exercise may decrease vasomotor symptoms is the fact that regular use of large muscle groups stimulates production of hypothalamic β-endorphin, which in turn affects the thermoregulation in the hypothalamus and makes it more stable. Also sex steroids stimulate hypothalamic production of β-endorphin but after menopause sex steroids decrease leading to lower stimulation of β-endorphins and a more labile thermoregulation (Hammar et al 1990; Wyon et al 1995). A recent not yet published American study found, according to a presentation at the meeting of the North American Menopause Society in 2012, no effects of exercise on vasomotor symptoms, but that study included mostly aerobic exercise using treadmill and cross-trainer.

Exercise and other health effects: Reports with various levels of evidence suggest that exercise acts both preventively and therapeutically in relation to a number of diseases such as cardiovascular disease, obesity, hypertension, osteoporosis, dementia, as well as breast cancer and some other cancers (McTiernan 2008, Erickson et al 2012). These effects may be due to reduced oxidative stress and inflammation caused by regular exercise. HRQoL, anxiety, daily stress symptoms and depressive symptoms have been shown to be positively affected by exercise (Rutanen 2013, Villaverde Gutierrez 2012).

Choice of mode of physical exercise: Physical exercise may be exerted in numerous different ways and it is important to define what kind of exercise is used in different studies, especially if the aim is to validate exercise as therapy. Since the effects of exercise on vasomotor symptoms may be caused by induction of hypothalamic endorphins in turn due to afferent signals from large activated muscle groups it seems reasonable to try resistance training as treatment of the vasomotor symptoms. Resistance training is also possible to individualize in relation to the subjects’ strength and strength gains. When testing muscular strength, the American College of Sports Medicine (ACSM, 2010) recommends tests of four or eight repetitions (4 Repetition Maximum =4RM or 8RM), when maximal strength tests cannot be done. This means that the subject finds the maximum load that she can lift four or eight times but not more. 8RM highly correlates with the gold standard isokinetic tests and has a test-retest reliability between 0.96-0.99. The 8RM test method also seems to be useful for finding the correct intensity of resistance training (Taylor & Fletcher 2012) and the test may be used as a part of the training session (ACSM 2010).

Exercise and effects on telomeres: Telomeres are repetitive sequences of DNA situated at the end of chromosomes of mammalians. They contribute to the function and stability of the chromosomes and are shortened during aging and by cell divisions (Eisenberg 2011). The length of the telomeres in mononuclear cells in peripheral blood has been suggested to be a marker of aging and shorter telomeres have been found to be associated with age-related diseases like cardiovascular disease, diabetes type 2, and dementia and also with increased mortality (Cawthon 2003, Mainous 2010, Tentolouris 2007). Estrogen may prevent shortening of telomeres probably by means of an antioxidative effect (Vina 2005) and by activation of the enzyme telomerase (Simoncini 2000, Grasselli 2008). Women who had used HT for a long time had longer telomeres than women who had not used HT (Lee 2005) and physically active women also had longer telomeres than sedentary women, although evidence is scarce (Du 2011, Kim 2012).

Exercise and effects on adipose tissue: White adipose tissue is specialized to store energy during periods with positive energy balance and to mobilize energy when needed. An excess of white adipose tissue causes obesity. Brown adipose tissue mainly produces heat and is most abundant in the new-born, but has recently been found to exist also in adults (Langin 2010). Brown adipose tissue contains active mitochondria which express uncoupling protein 1 (UCP1) important for the production of heat, but may also affect general metabolism, insulin sensitivity and weight changes (Lowell 1993, Yang 2003, Almind 2007). Even mature white fat cells may be converted to brown fat cells (Langin 2010). Physical activity has, mainly in mice, been shown to induce production of irisin, which can convert white fat cells to brown fat cells (Boström 2012). Thus physical activity may make adipose tissue more metabolically active and involved in heat regulation. These findings from animal studies, however, need to be tested and confirmed in human studies.

Exercise and mediators of inflammation: Both skeletal muscle and adipose tissue are endocrine organs, muscle tissue producing FNDC5, a membrane protein that is cleaved and secreted as the hormone irisin, upon exercise (Boström et al 2012). The latter is amply recognized as endocrine organ, which might present an altered function (as in obese patients) classed as systemic low-grade inflammation. Adipocytes from obese patients can for instance show an increased secretion of pro-inflammatory adipokines, such as TNF, MCP-1, DPP4 etc (Raschke & Eckel 2013). Moderate physical activity is associated with lower inflammatory activity for example measured as lower c-reactive protein (CRP) (Loprinzi et al, 2013), interleukin-6 and TNF (Beavers et al, 2010) whereas strenuous activity like marathon running may cause pro-inflammatory reactions (Bernecker et al, 2013). A recently published meta-analysis has confirmed that RT as intervention significantly decreases the levels of leptin (-40%), adiponectin (-10%) and of other inflammatory markers (CRP and interleukin (IL)-6) (Rodrigues Soares & Cordeiro de Sousa, 2013). Since the cardio-protective effects of exercise may be at least partially caused by lowered inflammatory activity it seems important to establish if a 15 weeks program of resistance training affects inflammatory status.

User perspective, psychological possibilities and obstacles when changing life-style with substantially increased physical activity: A clinically very relevant question is how to best stimulate people to change or amend life-style. In order to better understand the obstacles and possibilities when starting a long-term resistance training program when suffering from hot flushes we intend to explore the issue by using qualitative methodology and record interviews with a number of participants in the intervention group using individual or group interviews depending on the circumstances. We plan to perform these interviews with all women randomized to intervention, also those who are not compliant, in order to improve the handling of the patients over the study.

Originality, design and relation to the research frontier: The study is the first randomized, controlled, prospective study of a structured mode of physical exercise that is tailored to the women and measures a number of relevant health outcomes. It has been known since decades that there is a need for alternative therapies for women (and men) with vasomotor symptoms. We were the first worldwide to suggest and show that physical activity may benefit such symptoms but still it has remains to be proven that intervention utilizing exercise may affect the frequency and severity of symptoms and also what kind of exercise that is beneficial. Furthermore exercise may cause a number of long-standing health effects (e.g. decreased risk of cardiovascular disease, inflammatory diseases and cancer), but this has to be proven with strict scientific methods.

Project description: The theory behind our questions leading us to perform this project is that regular physical activity may cause substantial effects on the aging woman, by means of several mechanisms. These include afferent nervous signals affecting neurotransmitter production in the central nervous system, e.g. beta-endorphins and thereby in turn affecting thermoregulation, well-being, stress levels etc. Furthermore, probably by means of antioxidative effects, the risks of several age-related diseases are diminished, like cardiovascular disease, cancer etc. Metabolism and the conversion of white adipose tissue to brown adipose tissue may be further affected through production of myokines like irisin, with metabolic consequences for the aging woman. By unknown mechanisms also the immune system may be affected by physical activity.

The intention of the study: We intend to show that the intervention with regular resistance training (RT) will decrease the frequency and severity of vasomotor symptoms and thereby affect mental state and quality of life in postmenopausal women. We also intend to more deeply explore the mechanisms of RT on factors with long-term health effects on the aging woman, such as the inflammatory state (via monitoring specific adipo-myokines), body composition, and the relation between white and brown fat.

Material and methods: This is an open, prospective, randomized controlled intervention study with the decrease in number of hot flushes after 15 weeks intervention being the primary out-come and a number of secondary out-comes.

Patients: We intend to include sedentary women (maximum 75 minutes of intense exercise per week) who are postmenopausal, i.e. had their last menstrual bleeding at least 12 months ago or have increased gonadotrophic hormones showing they are postmenopausal. To be included they have to have at least 28 moderate to severe hot flushes per week during a screening period of two weeks.

The women will be randomized between intervention (supervised RT over 15 weeks) and a control group (“no change”). By means of a pilot study including 20 women we intend to find out how many women have to be included in order to be able to show a significant difference in change of the number of hot flushes/24 hours between baseline and 15 weeks. After this sample size calculation and addition of another 30% of women (due to a for-seen drop-out rate) this number of postmenopausal women with vasomotor symptoms will be randomized to either regular RT or “no change”. The RT will be supervised and individualized by an experienced physiotherapist first with lower relative load during three weeks based on individual baseline measurements of muscular strength. Thereafter the load will be increased over another 12 weeks.

Methods (see also flow chart): At baseline and after 3, 7, 11, and 15 weeks of intervention or observation we will measure frequency and severity of hot flushes/24 hours (diaries).

The following variables will be measured at baseline and after 15 weeks of intervention or “no change”:

a/ Health-related Quality of Life (Women’s Health Questionnaire; WHQ, Short Form Health Survey; SF-36)

b/ muscle strength (in arms and legs according to 8 repetition maximum tests; 8RM)

c/ level of physical activity (diary and International Physical Activity Questionnaires; IPAQ-scale; Craig 2003)

d/ concentrations of adipo-myokines/inflammatory biomarkers (Leptin, Irisin, IL-6, IL-7, IL-8, monocyte chemoattractant protein-1(MCP-1), tumor necrosis factor (TNF), IL-4, IL-10, IL-15, brain-derived neurotrophic factor (BDNF), matrix metalloproteinase (MMP)-2 and MMP-9 in blood samples shortly after a resistance-training and on a day without work-out; samples with direct measurement (plasma) and after white blood cell stimulation, conditioned medium with multiple bead technology (Luminex) and enzyme linked immunosorbent assay (ELISA)

e/ length of telomeres (in DNA isolated from blood samples with a qPCR based method (Pooley, 2010)). Recently, the telomere length was associated with certain SNP´s in the hTERT gene (Bojesen, 2013) and these will be assessed to control for genetic influence of telomere length.

f/ using magnetic resonance imaging (MRI) the volume of total (TAT), subcutaneous (SCAT) and visceral (VAT) white adipose tissue (Dahlqvist Leinhard 2008); the concentration of lipids diffusively stored in the liver parenchyma (Erlingsson 2009), and the volume of intramuscular adipose tissue (IMAT) in the quadriceps muscle (Gerdle 2013) will be quantified. Furthermore, the MRI images will be used to quantify the volume of and fat concentration in supraclavicular and interscapular brown adipose tissue according to our recently published measurement protocol (Lidell 2013, Borga 2013), as well as the volume of the major extensor and flexor muscles (Karlsson 2013).

g/ muscle mass, body mass index, abdominal height

h/ blood pressure.

Additionally, qualitative methodology will be used and a number of women will be interviewed in order to explore and identify facilitators as well as obstacles from changing/amending life-style with substantially increased physical activity.

At 6 and 24 months after end of the intervention and participation in the control group we plan to make a mailed follow-up using questionnaires and measurement of vasomotor symptoms (diary for 1 week), HRQoL (WHQ, SF-36) and exercise habits (IPAQ).

Time plan: The ethical application was approved during summer 2013 and we intend to start the pilot study by October 2013 and the full-sized study during fall 2014. From long-term experience with this patient group we know it will take long time to recruit women and hope to have completed the study by 2016-17 and will make the analyses and reports immediately after completion of the last patient.

The collaborating groups: The project will be performed in collaboration with a number of groups representing (a-g; see below) both clinical medicine (a,b,c), technology (c), basic biomedicine (c-f), and caring science (g).Without collaboration between these complementary groups this project would simply not be performed. This collaboration will bring a deeper understanding of the numerous expected effects we foresee from the intervention.

a/ Our own group from obstetrics and gynecology has long-term experience and competence from performing clinical intervention studies both pharmacological studies and studies with alternative therapies (acupuncture, physical activity, acupuncture, behavioral therapy). We consist of gynecologists and a very competent PhD-graduated research nurse, who today educates nation-wide clinical researchers on Good Clinical Practice. b/ We have a very competent physiotherapist, a PhD student who participated already during the design phase in order to plan and perform the intervention and measurements of muscular strength in the best possible way c/ For measurements of muscular and adipose tissue we collaborate with professors Magnus Borga and Olof Dahlqvist Leinhard at the Centre for Medical Imaging and Visualization (www.cmiv.liu.se), which is a successful collaboration between medicine and technology. d/ The immunological measurements will be performed together with professor Heriberto Rodriguez-Martinez (reproductive biologist) and his research fellow Marie Rubér (PhD) making translational research between cell-biology and clinics. e/ The irisin measurements will be performed by Pontus Boström, MD, PhD, from Karolinska Institutet, who was the first author of the original Nature paper on this peptide in spring 2012. f/ Telomere length and SNP measurements will be performed by professor Peter Söderkvist, a translational researcher within mainly genetics. g/ The qualitative studies including interviews will be performed in collaboration with professor Carina Berterö who is experienced and skilled in qualitative methodology as well as in mixed methods research.

Significance and relevance: It is of great importance to find alternatives to HT for vasomotor symptoms, which are very prevalent in women in general and even more prevalent in women with breast cancer and men with prostate cancer. Physical exercise has been found, mainly in cross-sectional studies, to be associated with less vasomotor symptoms but this has to be confirmed in prospective, controlled studies. It is also essential to find out which forms of exercise are effective. In this study, based on the hypothesis that engaging large muscle groups will have the greatest effects on hypothalamic beta-endorphin production and thus on the vasomotor symptoms, we focus on resistance training engaging large muscle groups. If we can find effects from the intervention in otherwise healthy women the method could easily be transferred to other patient groups like men with similar symptoms and to patients with breast and prostate cancer – and in the longer perspective to the aging population.

Furthermore, since regular exercise probably induces a number of beneficial health effects, it is important to broaden our understanding and knowledge of various effects from resistance training on mental, metabolic and immunologic functions. It is also important from a practical, clinical point of view to understand the possibilities and obstacles when substantially changing or amending life-style. Performing a qualitative interview-study with a number of the women could explore the issue and deepen the knowledge of how to succeed with the interventions.

We consider the study to be truly translational, involving biomedical methods and technology (such as modern imaging methodology) to gain new knowledge that can be transferred to clinical use and increase the health of the population. Thus involvement of several scientific areas and disciplines creates increased value both scientifically and for the society.

Clinical relevance and feasibility: This study may only be performed in collaboration with a clinical department and uses a number of available clinical facilities. Because the project group was early created with a mixture of clinically experienced and skilled personal and basic scientist the study has already during the pilot phase been shown to be feasible. If we can confirm previous findings from cross-sectional studies that physical exercise decreases vasomotor symptoms but also causes mental, metabolic and immunological effects this is a substantial support when counseling aging (and younger) patients with a number of diseases and as prevention for healthy people.

Implementation: If we can confirm our hypotheses and show both specific effects on the symptoms of the postmenopausal woman and the general health benefits the intervention may easily be implemented into clinical practice. There is already a tradition within Swedish health care to prescribe physical exercise when proven to be evidence based.

Preliminary results: Already in 1990 (as the first group worldwide) we reported that vasomotor symptoms were less prevalent in women who participated in regular physical exercise compared with two age-cohorts of women from the same geographic area (Hammar et al 1990) which we confirmed in a later cross-sectional study (Ivarsson et al 1998) and women who were randomized to, and were compliant to, intervention with regular exercise reported decreased vasomotor symptoms and increased HRQoL (Lindh-Åstrand et al, 2004). This latter study was the only that was included in the first Cochrane analysis on exercise and vasomotor climacteric symptoms. We have also performed numerous intervention studies on women and men with vasomotor symptoms, both otherwise healthy and with a malignant disease (breast and prostate cancer). The present planned study is based on experience from our previous intervention studies but involves a number of outcomes that we have yet not measured in relation to physical exercise. The pilot part of the study is ongoing and we have already screened about 20 women and randomized 15 of them to intervention or “no change”.

User perspective: psychological possibilities and obstacles when changing life-style with substantially increased physical activity: A clinically very relevant question is how to best stimulate people to change or amend life-style. In order to better understand the obstacles and possibilities when starting a long-term resistance training program when suffering from hot flushes we intend to explore this important issue. We intend to use qualitative methodology and record interviews with a number of participants in the intervention group using individual or group interviews depending on the circumstances. We plan to perform these interviews with all women randomized to intervention, also those who are not compliant, in order to improve the handling of the patients over the study. In this way we clearly involve the “user” in evaluating the methods used and intend to collect experiences and impressions from the women involved in the study, with probable impact on future implementation of the results.

Collaboration; national and international collaboration, gender and age: We have performed international and national collaborations related to industry sponsored pharmacological studies and collaboration on effects of acupuncture on vasomotor symptoms in women (Borud et al 2009; Spetz Holm et al 2013). In the present study we have active collaboration with several research groups from Linköping University and with Karolinska Institutet. Several of the collaborating groups have numerous international collaborators not mentioned here. The research group includes senior as well as more junior collaborators of both sexes and representing different areas and disciplines.

The project involves and is totally dependent upon collaboration between research groups from clinical medicine, caring science, physiotherapy, science and technology, genetics and basic biomedicine.

Other grants: We have this far not applied for other grants than the present and the Family Kamprad foundation, except ALF-grants from the County Council of Östergötland and internal grants from Linköping University (strategic grants after my 6 years as a dean). The available equipment (see below) is financed by grants from the university and other external sources.

Equipment: The project is based on equipment available at the respective universities. The MRI examinations will performed on a state of the art 3.0 T Philips Ingenia (Philips Medical System, Best the Netherlands) MR-scanner at the Center for Medical Image Science and Visualization (CMIV) at Linköping University.

Need for infrastructure: We need a Luminex 200 reader for the immunological analyses.

Ethical and related consideration: The study will be performed according to Good Clinical Practice and the latest version of the Helsinki Declaration. It will be monitored by an independent monitor from Linköping Academic Research Center. The study has been approved by the Regional Ethical Committee in Linköping (Dnr 2013/285-31).

The risks with the intervention are related to the resistance training. The risk of overuse injuries is minimized by the supervision and gradually increased load. Women may report problems at every visit to the training center (three times/week) and visits to the clinic, or may call us daily. The risks with blood sampling are minor and mostly discomfort. The MR examination causes no radiation but could cause discomfort like claustrophobia. Skilled staff who are in contact with the woman minimize this risk.

The advantages include the health promoting effects from changing life-style for women in the intervention group. Women randomized to “no change”, i.e. the control group, will be offered instruction about resistance training and free-of-charge training at the training center for a period after completion of the 15 weeks. On the population level the advantages are the possibility to validate resistance training as a treatment method for middle-aged women with vasomotor symptoms and in the longer perspective other patient groups.

Every participant is informed both in writing and orally that she may withdraw her participation at any time without negative consequences on future care. All women are asked to fill in an informed consent before anything related to the study is performed.

A clinically very relevant question is how to best stimulate people to change or amend life-style. In order to better understand the facilitators and barriers when starting a long-term resistance training program we intend to use qualitative methodology to explore this issue. Via recorded interviews with a number of participants in the intervention group we are able to present their point of view - perhaps the issue is about amending their life-style, not totally changing it. We intend to interview the first 20 women randomized to the intervention, both those who are compliant and those who are not, in order to improve routines during the study.

References:

Albertazzi P. Non-estrogenic approaches for the treatment of climacteric symptoms.

Almind K, Manieri M, Sivitz W et al Ectopic brown adipose tissue in muscle provides a mechanism for differences in risk of metabolic syndrome in mice. Proc Natl Acad Sci USA 2007;104:2366-71

Beavers KM, Brinkley TE, Nicklas BJ. Effect of exercise training on chronic inflammation. Clin Che Acta 2010:411:785-793.

Bernecker C, Scherr J, Schinner S, et al. Evidence for an exercise induced increase of TNF-a and IL-6 in marathon runners. Scand J Med Sci Sports 2013;23:207-214.

Borga M., Virtanen Kirsi A., Romu T., Dahlqvist Leinhard O. Persson A., Nuutila P., Enerbäck S. Brown adipose tissue in humans: detection and functional analysis using PET (Positron Emission Tomography), MRI (Magnetic Resonance Imaging), and DECT (Dual Energy Computed Tomography), accepted for publication in: Methods in Enzymology Volume 537: Methods of Adipose Tissue Biology.

Bojesen SE et al., Multiple independent variants at the TERT locus are associated with telomere length and risks of breast and ovarian cancer Nature Genetics, 45,371–384, (2013).

Borud E, et al. The Acupuncture on hot flushes among Menopausal Women (ACUFLASH) study, a randomized controlled trial. Menopause, 2009; 16:484-93

Boström P, Wu J, Jedrychowski MP, et al. A PGC1-α-dependent myokine that drives brown-fat-like development of white fat and thermogenesis. Nature. 2012 Jan 11;481(7382):463-8.

Cawthon RM, Smith KR, O'Brien E, et al. Association between telomere length in blood and mortality in people aged 60 years or older. Lancet. 2003 Feb 1;361(9355):393-5

Cohen SM, Rosseau ME, Carey BL. Can acupuncture relieve symptoms of the menopause? Holist Nurs Pract 2003;17:295-9

Craig C L, Marshall A L, Sjöström M, et al. International Physical Activity Questionnaire: 12-country reliability and validity. Medicine & Science in Sports & Exercise. 2003;35(8):1381-1395.

Dahlqvist Leinhard O, Johansson A, Rydell J et al. Quantitative Abdominal Fat Estimation Using MRI. 2008 19th International Conference on Pattern Recognition, ICPR 2008 , art. no. 4761764.

Daley A, Stokes-Lampard H, Macarthur C. Exercise for vasomotor menopausal symptoms. Cochrane Database Syst Rev. 2011 May 11;(5):CD006108. doi:10.1002/14651858.CD006108.pub3. Review

Du M, et al. Physical activity, sedentary behavior, and leukocyte telomere length in women. Am J Epidemiol. 2012 Mar 1;175(5):414-22.

Eisenberg DT. An evolutionary review of human telomere biology: the thrifty telomere hypothesis and notes on potential adaptive paternal effects. Am J Hum Biol. 2011 Mar-Apr;23(2):149-67

Erickson KI, Weinstein AM, Lopez OL. Physical activity, brain plasticity, and Alzheimer's disease. Arch Med Res. 2012 Nov;43(8):615-21

Gerdle B, Forsgren MF, Bengtsson A, et al. Decreased muscle concentrations of ATP and PCR in the quadriceps muscle of fibromyalgia patients - a 31P MRS study. Epub in European Journal of Pain, 30 JAN 2013 DOI: 10.1002/j.1532-2149.2013.00284

Grasselli A et al. Estrogen receptor-alpha and endothelial nitric oxide synthase nuclear complex regulates transcription of human telomerase. Circ Res. 2008 Jul 3;103(1):34-42.

Hammar M, Berg G, Lindgren R (1990) Does physical exercise influence the frequency of postmenopausal hot flushes?, Acta Obstet. et Gynecol. Scand 69:409

IPAQ (http://sites.google.com/sites/theipaq/ )

Ivarsson T, Spetz A-C, Hammar M (1998) Physical exercise and vasomotor symptoms in postmenopausal women, Maturitas 29:139

Karlsson A et al. Whole Body Muscle Classification using Multiple Prototype Voting. Proceedings of the ISMRM Annual Meeting (ISMRM'13), 2013.

Kechagias S, Ernersson A, Dahlqvist O, et al. Fast-food-based hyper-alimentation can induce rapid and profound elevation of serum alanine aminotransferase in healthy subjects. Gut. 2008 ;57:649-54.

Kim JH, Ko JH, Lee DC, Lim I, Bang H. Habitual physical exercise has beneficial effects on telomere length in postmenopausal women. Menopause. 2012 Oct;19(10):1109-15

Langin D. Recruitment of brown fat and conversion of white into brown adipocytes: strategies to fight the metabolic complications of obesity? Biochim Biophys Acta. 2010 Mar;1801(3):372-6.

Lee DC et al. Effect of long-term hormone therapy on telomere length in postmenopausal women. Yonsei Med J. 2005 Aug 31;46(4):471-9.

Lidell M, et al. Evidence for Two Types of Brown Adipose Tissue in Humans. Nature Medicine, Volume 19(5):631-634, 2013, DOI:10.1038/nm.3017.

Lindh-Astrand et al.. Vasomotor symptoms and quality of life in previously sedentary postmenopausal women randomised to physical activity or estrogen therapy Maturitas; 2004; 48: 97-105

Loprinzi et al. Objectively measured physical activity and C-reactive protein: Natioal Health and Nutrititon Examination Survey 2003-2004. Scand J Med Sci Sports 2013;23:164-170.

Lowell BB, Susulic V, Hamann A, et al. Development of obesity in transgenic mice after genetic ablation of brown adipose tissue. Nature 1993;366:740-742

Maclennan AH, Broadbent JL, Lester S, Moore V. Oral oestrogen and combined oestrogen /progestogen therapy versus placebo for hot flushes. Cochrane Database Syst Rev 2004(4)

Mainous AG et al. Leukocyte telomere length and coronary artery calcification. Atherosclerosis. 2010 May;210(1):262-7.

McTiernan A. Mechanisms linking physical activity with cancer. Nat Rev Cancer. 2008;8(3):205-11

Nappi C, et al. Relationship between cerebrospinal fluid beta-endorphin and plasma pituitary-gonadal hormone levels in women. J Endocrinol Invest. 1990; 2:149-53.

Nedstrand E, Wijma K, Wyon Y, Hammar M. Vasomotor symptoms decrease in women with breast cancer randomized to treatment with applied relaxation or electro-acupuncture: a preliminary study. Climacteric 2005;8:243-50.

Pooley KA, et al. Telomere length in prospective and retrospective cancer case-control studies. Cancer Res., 2010 Apr 15;70(8):3170-6.

Raschke S & Eckel J (2013) Adipo-myokines: two sides of the same coin – mediators of inflammation and mediators of exercise. Mediators of inflammation 2013, article ID 320724 , 16 pages, Hindawi Publ Corp (doi: 10.1155/2013/320724.

Rodrigues Soares FH & Cordeiro de Sousa MB (2013) Different types of physical activity on inflammatory biomarkers in women with or without metabolic disorders: a systematic review. Women & Health 53: 298-316.

Rossouw JE, Anderson GL, Prentice RL, et al, Beresford SA, et al: principal results From the Women's Health Initiative randomized controlled trial. JAMA. 2002 Jul 17;288(3):321-33.

Rutanen et al. Effect of physical exercise on work ability and daily strain in symptomatic menopausal women: A randomized controlled trial. Work. 2013 Jan 15. [Epub ahead of print

Shen W and Stearns V. Treatment strategies for hot flushes. Expert opin. Pharmacother. 2009 10(7):1133-1144.

Simoncini T et al. Interaction of oestrogen receptor with the regulatory subunit of phosphatidylinositol-3-OH kinase. Nature. 2000 Sep 28;407(6803):538-41.

Taylor J D, Fletcher J P. Reliability of the 8-repetition maximum test in men and women. Journal of Science in Medicine and Sports. 2012;15:69-73.

Tentolouris N, Nzietchueng R, Cattan V, et al. White blood cells telomere length is shorter in males with type 2 diabetes and microalbuminuria. Diabetes Care. 2007 Nov;30(11):2909-15.

US Department of Health and Human Services. 2008 Physical Activity Guidelines for Americans: Be Active, Healthy, and Happy! Washington DC: US Department of Health and Human Services; 2008.

Villaverde Gutiérrez C, Torres Luque G, et al.Influence of exercise on mood in postmenopausal women. J Clin Nurs. 2012 Apr;21(7-8):923-8. doi: 10.1111/j.1365-2702.2011.03972.x.

Viña J, Borrás C, Gambini J, Sastre J, Pallardó FV. Why females live longer than males? Importance of the upregulation of longevity-associated genes by oestrogenic compounds. FEBS Lett. 2005 May 9;579(12):2541-5.

Wyon et al. Effects of acupuncture on climacteric vasomotor symptoms, quality of life and urinary excreation of neuropeptides among postmenopausal women, Menopause 1995;2:3-12.

Wyon Y, Wijma K, Nedstrand E, Hammar M. A comparision of acupuncture and oral estradiol treatment of vasomotor symptoms in postmenopausal women. Climacteric 2004;7:153-64.

Yang X, Enerback S, Smith U. Reduced expression of FOXC2 and brown adipogenic genes in human subjects with insulin resistance. Obes Res 2003;11:1182-1191

Table showing the plan of the 2 year follow-up study after the initial 15 week intervention study

Visit no: 1

Ob-gyn 2

Ob-gyn 3 4 5

6

Ob-gyn Follow up

Telphone/

Questionnaire/Visit

Week no: → -2

Screen 0

BL 3 7 11 15 6 months 24 months

Informed consent X

Interview (IG) X

Medical history X

Physical examination X

Weight, length (BMI) X X X

Blood pressure X X X

Incl/exclusion criteria X X (for the 2 years follow-up study)

Blood sampling (Hemoglobin)

FSH (if necessary) X

(X) X

Bloodsampling (telomeres, adipo/myokines) X X X

Randomization X

HRQoL (WHQ, SF-36) X X X X

Muscle strength X X IG X

IPAQ questionnaire X X X X

Diary (hot flushes) X X X X X X X

MR X X X

Health problems, other therapies X X X

IG: Intervention group, CG; control group, Scr; Screening BL; baseline
